# DNA methylation and transcriptome analysis reveal epigenomic differences among three macaque species

**DOI:** 10.1111/eva.13604

**Published:** 2023-10-12

**Authors:** Jiao Wang, Xuyuan Liu, Yue Lan, Tengcheng Que, Jing Li, Bisong Yue, Zhenxin Fan

**Affiliations:** ^1^ Key Laboratory of Bioresources and Eco‐Environment (Ministry of Education), College of Life Sciences Sichuan University Sichuan Chengdu China; ^2^ Sichuan Key Laboratory of Conservation Biology on Endangered Wildlife, College of Life Sciences Sichuan University Sichuan Chengdu China; ^3^ Terrestrial Wildlife Rescue and Epidemic Diseases Surveillance Center of Guangxi Guangxi Nanning China; ^4^ Faculty of Data Science City University of Macau Macau Taipa China

**Keywords:** DNA methylation, epigenetics, gene expression, *Macaca*, neuro development

## Abstract

Macaques (genus *Macaca*) are the most widely distributed non‐human primates, and their evolutionary history, gene expression profiles, and genetic differences have been extensively studied. However, the DNA methylomes of macaque species are not available in public databases, which hampers understanding of epigenetic differences among macaque species. Epigenetic modifications can potentially affect development, physiology, behavior, and evolution. Here, we investigated the methylation patterns of the Tibetan macaque (*M. thibetana*; TM), Chinese rhesus macaque (*M. mulatta lasiota*; CR), and crab‐eating macaque (*M. fascicularis*; CE) through whole‐genome bisulfite sequencing from peripheral blood. We compared genome‐wide methylation site information for the three species. We identified 12,128 (CR vs. CE), 59,165 (CR vs. TM), and 39,751 (CE vs. TM) differentially methylated regions (DMRs) in the three macaques. Furthermore, we obtained the differentially expressed genes (DEGs) among the three macaque species. The differences between CR and CE were smaller at both the methylome and transcriptome levels than compared with TM (CR vs. TM and CE vs. TM). We also found a change in the density of single nucleotide mutations in DMRs relative to their flanking regions, indicating a potential mechanism through which genomic alterations may modulate methylation landscapes, thereby influencing the transcriptome. Functional enrichment analyses showed the DMR‐related genes were enriched in developmental processes and neurological functions, such as the growth hormone‐related pathway, insulin secretion pathway, thyroid hormone synthesis pathway, morphine addiction, and GABAergic synapses. These differences may be associated with variations in physiology and habitat among the macaques. Our study provides one of the first genome‐wide comparisons of genetic, gene expression, and epigenetic variations across different macaques. Our results should facilitate further research on comparative genomic and genetic differences in macaque species.

## 1 INTRODUCTION

DNA methylation is the main mode of epigenetic modification, which can alter genetic expression without altering the DNA sequence and can silence genes, making them ineffective. As an essential gene regulatory process, DNA methylation facilitates phenotypic variability in response to environmental change, independent of genetic variation (Angers et al., 2010; Jeremias et al., 2018). Furthermore, DNA methylation plays a significant role in organismal development, gene expression profiles, genomic imprinting, disease susceptibility, and evolutionary dynamics (Mendizabal et al., 2016). Epigenetic mechanisms can easily be integrated into a model of phenotypic variation in multicellular organisms, offering insights into the phenotypic differences observed among genetically identical organisms (Wong et al., 2005). While genetic disparities alone cannot fully account for observed phenotypic differences, research has demonstrated that DNA methylation can contribute to such variations. A classic example occurs in queen and worker bees, which share the same genome but exhibit different phenotypes. Notable: Lyko et al. (2010) reported significant differences in methylation levels in 500 genes between queen and worker bees, while Kucharski et al. (2008) used RNA interference to silence the *DNMT3* gene, resulting in the development of young bees into queens. DNA methylation is also associated with phenotypic differences in human populations (Heyn et al., 2013), especially among twins (Fraga et al., 2005; Kaminsky et al., 2009). Hence, DNA methylation serves as a mechanism capable of elucidating phenotypic disparities and providing a theoretical foundation for such observations.

Macaques, classified as endangered animals in their natural habitat, serve as important model animals in medical‐related research (Gibbs et al., 2007; Xu et al., 2022). Despite their similar genetic backgrounds, phenotypic differences exist among macaque species (Gibbs et al., 2007). Tibetan macaques (*Macaca thibetana*), which are endemic to China, differ substantially from crab‐eating macaques (*M. fascicularis*) in geographical range, body size, morphology, physiology, and behavior (Fan et al., 2014). Furthermore, compared with Chinese rhesus macaques (*M. mulatta lasiota*), TM exhibits a larger body size, a longer life span, a calmer temperament, and greater trainability (Solari & Baker, 2007). Previous analyses have identified the pattern of DNA differences among different macaques (Fan et al., 2014; Fan et al., 2018). For example, Fan et al. (2014) reported that Tibetan macaques possess more nonsynonymous variants in genes related to immune response and glucose metabolism than rhesus and crab‐eating macaques. Studies have demonstrated that DNA methylation levels differ among species (Klughammer et al., 2023). However, no studies have focused on the DNA methylation levels between different macaques and their correlation with gene expression levels. Thus, the small number of fixed genetic divergences at the DNA level may be insufficient for explaining all phenotypic differences. Consequently, there is an urgent need for further DNA methylation research.

To date, investigations into methylation patterns have included various primate species (Hernando‐Herraez, Garcia‐Perez, et al., 2015; Hodges et al., 2011; Molaro et al., 2011). However, the epigenomic differences among macaque species remain unclear. In this study, we used the latest high‐quality rhesus macaque reference genome (*M. mulatta*, Mmul_10) (Warren Wesley et al., 2020) and performed whole‐genome bisulfite sequencing (WGBS) on multiple *M. mulatta lasiota*, *M. fascicularis*, and *M. thibetana* individuals (with relationship (*M. thibetana* (*M. mulatta lasiota*, *M. fascicularis*))). We aimed to explore their epigenomic differences and investigate the relationship between epigenomic regulation and gene expression, as well as the association between genomic and epigenomic differences. This study should enhance our understanding of comparative genomics and genetic discrepancies within macaque species, thereby deepening our understanding of macaque health, disease, development, and more.

## 2 RESULTS

### 2.1 Genome‐wide DNA methylation landscape of three macaque species

A total of 21 transcriptomes and 15 methylomes (from two different groups of macaques; see Table S1) were sequenced or downloaded from previous studies on three macaque species, including Tibetan macaques (*M. thibetana*; TM), Chinese rhesus macaques (*M. mulatta lasiota*; CR), and crab‐eating macaques (*M. fascicularis*; CE). The bisulfite conversion rate of all samples was greater than 99% (Table S2). All methylome data were mapped to the *M. mulatta* genome (Mmul_10), with a mean efficiency of 75.75% (Table S2). After using deduplicate_bismark, the remaining 86.65%–97.60% of reads were used for subsequent analysis (Table S3). At the genome‐wide level, 2.92%–4.24% of cytosine sites were found to be methylated. Of these methylated sites, 82.45%–90.52% were in the mCpG context, 2.25%–4.44% in the mCHG context, and 7.23%–13.11% in the mCHH context. The overall DNA methylation patterns showed comparable genome‐wide methylation ratios among the different species, with 68.80%–75.50% of cytosine sites in the CpG context being methylated and less than 1% of cytosine sites in the CHG and CHH contexts being methylated (Figure S1a; Table S3). Methylation level distribution analysis demonstrated that the methylation levels at the CpG site were relatively high, with relatively low methylation levels at the CHH and CHG sites (Figure S1b).

We delineated trends in the methylation levels of gene bodies and flanking regions (Figure S1c). Notably, the region proximal to the transcription start site (dotted line on the left) showed significant fluctuations in methylation levels, gradually decreasing toward the transcription start site. In particular, CR showed a significant reduction in methylation levels near the transcription start site. Circos plots of mean methylation levels (Figure S2) showed both hypermethylated and hypomethylated regions in each chromosome of the three species. In addition, centromeres and their flanking regions exhibited relatively low levels of methylation.

**FIGURE 1:**
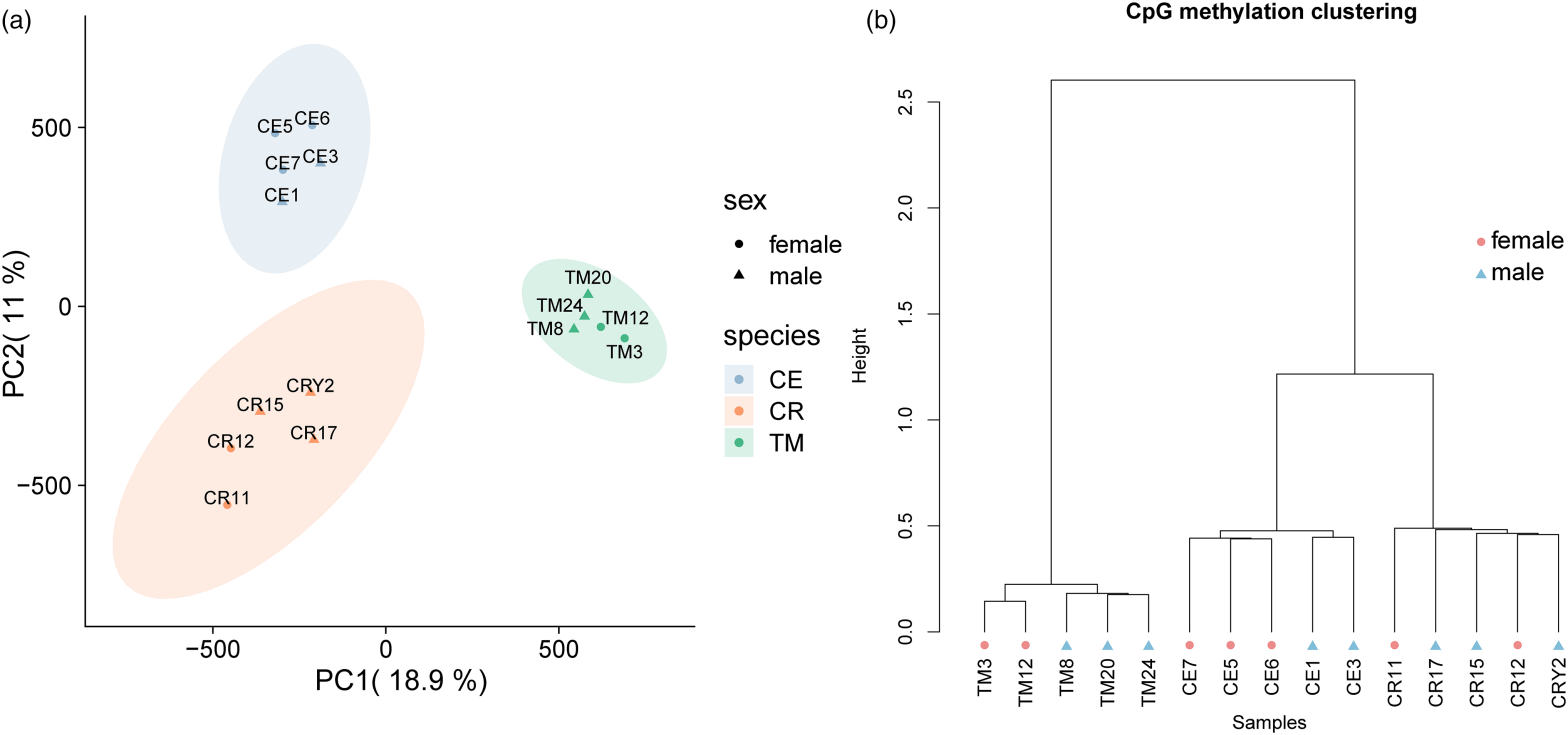
Display of overall methylation pattern and cluster analysis of CpG locus information. (a) PCA of CpG site methylation information. (b) Cluster analysis performed based on the methylation information of CpG sites. Distance method: “correlation”; Clustering method: “ward”.

To examine the factors shaping the distribution patterns of CpG methylation, we conducted principal component analysis (PCA) using the methylation data from the samples. As shown in Figure 1a, the two main components affecting methylation distribution accounted for 18.9% and 11% of the variance, respectively. The samples were also clustered by species. Thus, to clarify the methylation relationship among the three species, we performed cluster analysis of the CpG information of the samples (Figure 1b). The results were aligned with the PCA findings, confirming that samples from the same species formed distinct clusters, with a particularly close correlation between CR and CE.

### 2.2 Differentially methylated regions (DMRs) between different macaque species

**FIGURE 2:**
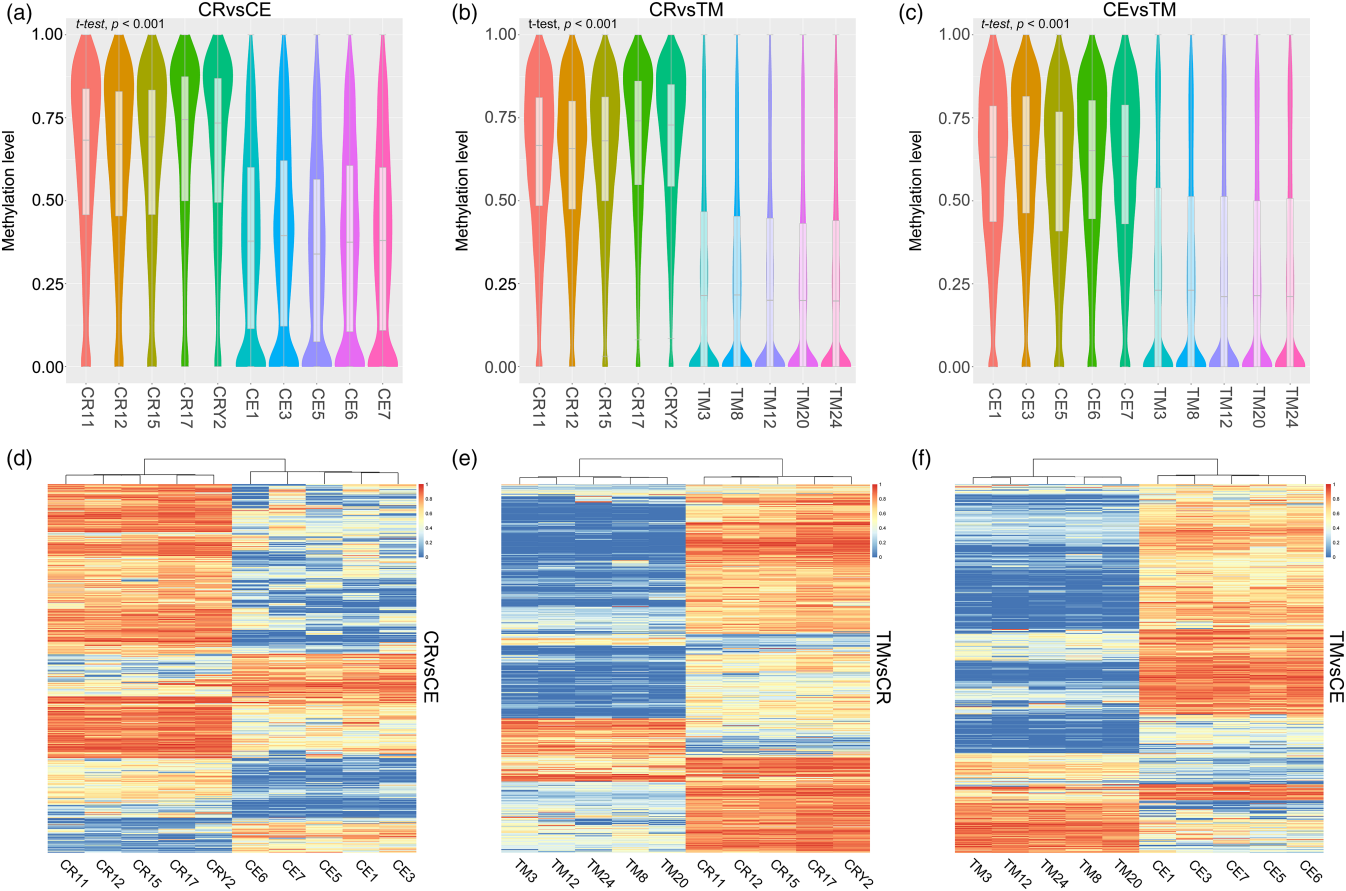
Methylation levels of differential methylation regions (DMRs). (a–c) The percent methylation levels of DMR in groups (a) CR versus CE, (b) CR versus TM, and (c) CE versus TM were plotted. “*t*‐test, *p* < 0.001” means that methylation levels differ significantly between any two macaque species.(d–f) The DMR heatmap of group (d) CR versus CE, (e) CR versus TM, and (f) CE versus TM. Each heatmap shows 2000 DMRs randomly selected from each group of DMRs.

Three groups of DMRs were identified from the 20 auto‐chromosomes: that is, CR versus CE (total *n* = 12,128; hypermethylated DMRs *n* = 5887; hypomethylated DMRs *n* = 6241; Table S4), CR versus TM (total *n* = 59,165; hypermethylated DMRs *n* = 42,834; hypomethylated DMRs *n* = 16,332), and CE versus TM (total *n* = 39,751; hypermethylated DMRs *n* = 30,338; hypomethylated DMRs *n* = 9413). In each group, hypermethylation and hypomethylation refer to the former species relative to the latter. The CR versus CE group exhibited a higher number of hypomethylated DMRs, whereas the CR versus TM and CE versus TM groups had a greater number of hypermethylated DMRs. However, for most DMRs, the mean methylation level was higher in CR than in CE and TM, and higher in CE than in TM (Figure 2a–f). We also excluded the effect of age‐ and sex‐related DMR on inter‐species DMR and found minimal overlap between age‐ and sex‐related DMR and inter‐species DMR, including only 0.1513% (168/111044) age‐ and 0.3899% (433/111044) sex‐related common DMRs with inter‐species DMRs (see Materials and Methods, Tables S4–S8).

**FIGURE 3:**
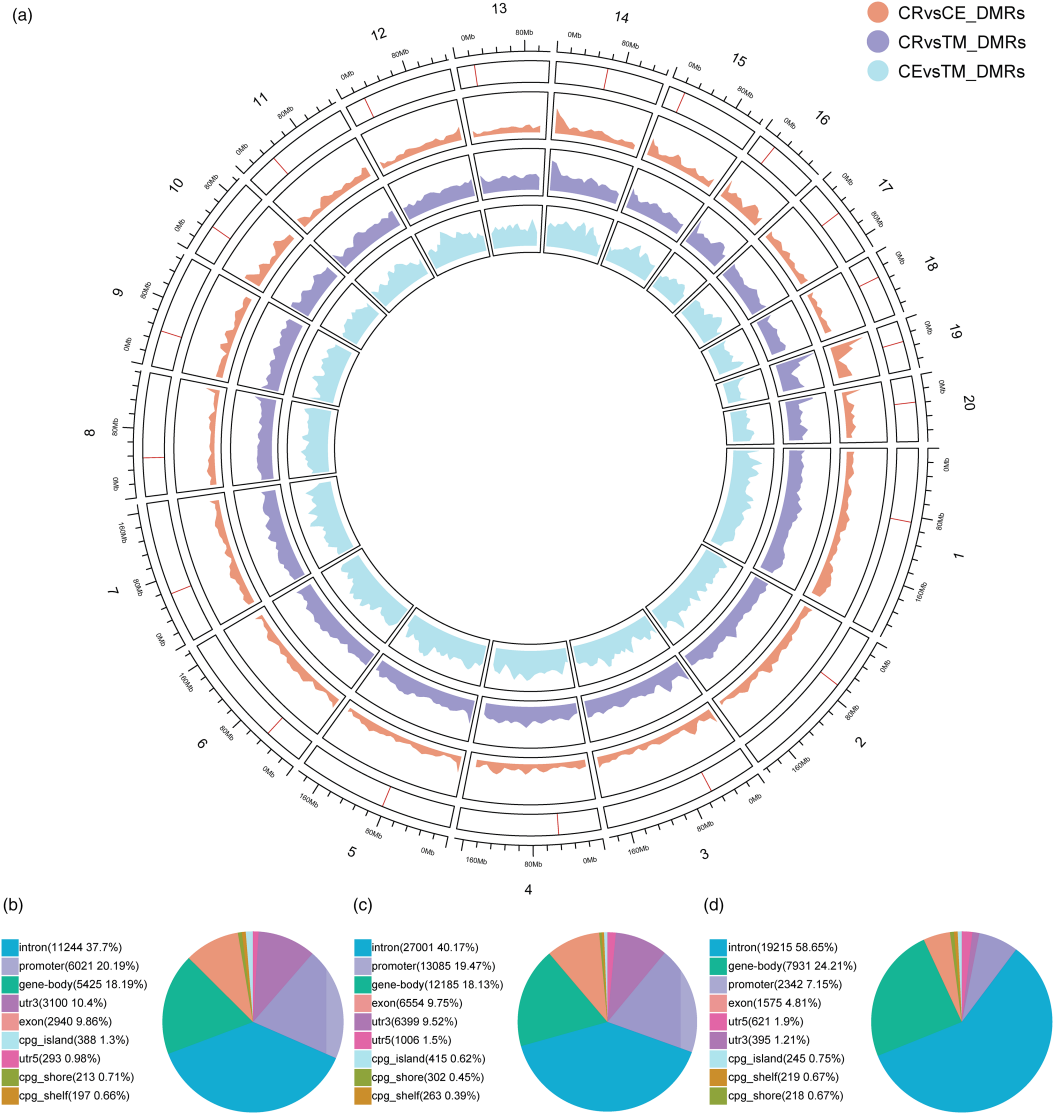
Distribution pattern of DMRs. (a) Circos plot of DMR density distribution. Different color areas in the figure represent the density distribution of DMRs in different groups. The red line in the outermost box represents the centromere. (b–d) The percentage of genomic regions overlapped with DMR. The different DMR groups from (b) to (d) are CR versus CE, CR versus TM, and CE versus TM.

To further analyze the distribution of methylation differences across the genome, we visualized the lengths of the DMRs (Figure S3). Interestingly, the DMRs in the three groups exhibited similar length distribution patterns (CR vs. CE: length range 51–8920 bp, average length 272.86 bp; CR vs. TM: length range 51–4995 bp, average length 278.40 bp; CR vs. CE: length range 51–4994 bp, average length 253.53 bp). At the chromosome level, Chromosome 1 had the largest number of DMRs in all three groups (1119 in the CR versus CE; 5054 in the CR versus TM; 3302 in the CE versus TM groups) (Figure S4). To determine the distribution of DMR density in each chromosome, we divided the length of the DMR on each chromosome by the total length of the chromosome (Figure S5). Results showed that the DMRs in the CR versus CE and CR versus TM groups reached maximum distribution density on chromosome 19 (CR vs. CE = 0.2696%; CR vs. TM = 0.897%). However, the highest DMR distribution density in the CE versus TM group was on chromosome 4 (0.4122%). The lowest DMR density in the CR versus CE and CR versus TM groups was on chromosome 13 (CR vs. CE = 0.0877%; CR vs. TM = 0.5533%). Interestingly, the lowest DMR density distribution in the CE versus TM group was on chromosome 19 (0.3082%). We also plotted the density distribution of DMRs and corresponding regions at the chromosomal level (Figure 3a). First, the distribution of DMRs in each chromosome was not uniform but fluctuated, with some chromosomal regions showing high DMR density. For example, the start of chromosomes 14 and 19 exhibited a higher DMR density, suggesting that differential methylation between species may prefer to occur in specific regions.

Next, we annotated functional regions of DMRs according to annotation files and counted the number of different genomic elements that overlapped with the DMRs (Figure 3b–d). Among the genomic elements, the proportion of introns was highest in all three groups: CR versus CE *n* = 11,244 (37.7%), CR versus TM groups *n* = 27,001 (40.17%), and CE versus TM *n* = 19,215 (58.65%). The CE versus TM group contained fewer overlapping promoters with DMRs (2342, 7.15%) compared to the other two groups. In addition, the proportion of CpG islands and flanking‐related areas covered by DMRs was small (0.39%–1.3%). Analysis of the coverage of repeating elements and DMRs indicated a high proportion of coverage over long and short‐interspersed nuclear elements (LINEs and SINEs) (Figure S6).

Finally, we obtained the DMR‐related genes (DMGS, overlapping with gene body) and DMR‐related promoters (DMPs, overlapping with promoters) in the three groups and found more common DMGs (2947) than common DMPs (244) among the groups (Figure S7). To better understand the biological functions of DMGs and DMPs, we conducted Gene Ontology (GO) and Kyoto Encyclopedia of Genes and Genomes (KEGG) enrichment analyses. Based on GO enrichment analysis of DMGs (Figure S8a–c; Table S9), negative regulation of cell communication (GO:0010648), cell projection morphogenesis (GO:0048858), and peptidyl‐amino acid modification (GO:0018193) were enriched in the CR versus CE group, cellular macromolecule catabolic processes (GO:0044265) and cellular protein catabolic processes (GO:0044257) were enriched in the CR versus TM group, and small GTPase‐mediated signal transduction (GO:0007264) was enriched in the CE versus TM group. Furthermore, overlapping DMGs (common in three macaques) were enriched in categories related to neuro development, including neuron development (GO:0048666), axon development (GO:0061564), and dendrite development (GO:0016358), and categories related to cell development, including cell morphogenesis (GO:0000902), cell projection morphogenesis (GO:0048858), and cellular component morphogenesis (GO:0032989). In the KEGG enrichment analysis, we plotted the 20 most significant pathways in each group (Figure S9a–c; Table S10). Several enriched KEGG pathways were related to essential hormones in organisms, including, but not limited to, growth hormone synthesis, secretion, and action (KEGG:04935), the insulin signaling pathway (KEGG:04910), and the thyroid hormone signaling pathway (KEGG:04919). We selected the *SOCS1* gene (*ENSMMUG00000010956*), which is involved in the regulation of physiological functions, to analyze the coverage of DMRs on genes. We analyzed the methylation level of *SOCS1* and found a significantly higher methylation level in one region of the gene in the TM group compared to the CR and CE groups. Moreover, the related DMR in the CR versus TM group (chr20: 11220602–11,221,998, diff.Methy = −0.349173575, *p* < 0.01) covered *SOCS1* (chr20: 11220610–11,221,257, reverse chain). The related DMR in the CE vs TM group (chr20: 11220711–11,221,349, diff.Methy = −0.317753274, *p* < 0.01) also covered most regions of the *SOCS1* gene.

Analysis of DMPs showed that only the CR vs CE and CR vs TM groups were enriched in GO categories and KEGG pathways. Based on GO enrichment analysis, both groups were enriched in actin filament‐based processes (GO:0030029), the Wnt signaling pathway (GO:0016055), and actin cytoskeleton organization (GO:0030036) (Figure S10a–b; Table S11). Based on KEGG pathway enrichment analysis, both groups were enriched in the Rap1 signaling pathway (KEGG:04015), growth hormone synthesis, secretion, and action (KEGG:04935), and axon guidance (KEGG:04360) (Figure S11a,b; Table S12). DMPs in the CE vs TM group and shared among all three groups showed no significant enrichment.

### 2.3 Gene expression analysis

**FIGURE 4:**
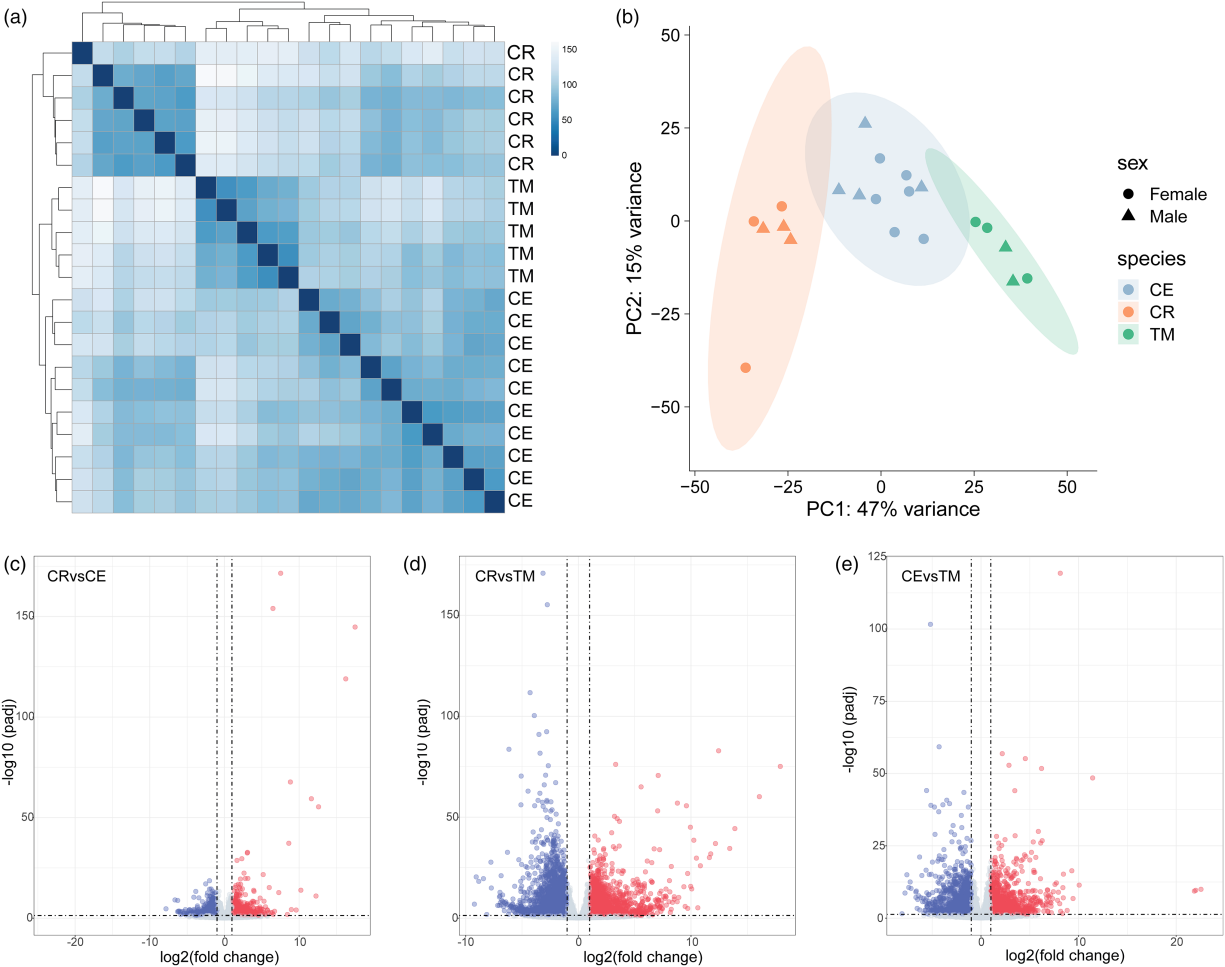
Gene expression profiles of three rhesus species. (a) Results of cluster analysis of gene expression profiles in transcriptome samples. (b) Results of PCA of gene expression profiles in transcriptome samples. (c–e) The species‐specific DEGs of (c) CR versus CE, (d) CR versus TM, and (e) CEvsTM. The genes marked in the figure are DEGs with LogFC >10.

Several studies have investigated gene expression differences at different physiological stages within specific *Macaca* species (Lan et al., 2020; Yan et al., 2020). However, research on gene expression differences between macaque species is scarce. Given the potential regulatory role of DNA methylation in gene expression, we examined the gene expression profiles of the three macaque species. Cluster analysis showed that samples were primarily clustered according to species (Figure 4a). Similarly, PCA demonstrated that the samples were mainly distributed according to species, with PC1 accounting for 47% of the variance, representing a major factor in species variation (Figure 4b). The distance between CR and CE was closer than that between CR and TM (Figure 4b). We next analyzed and identified three groups of differentially expressed genes (DEGs, |log2Fold‐Change| > 1 and FDR <0.05), namely, CR versus CE (total *n* = 1677, up‐regulated DEGs *n* = 820, down‐regulated DEGs *n* = 857), CR versus TM (total *n* = 5162, up‐regulated DEGs *n* = 2371, down‐regulated DEGs *n* = 2791), and CE versus TM (total *n* = 2895, up‐regulated DEGs *n* = 1245, down‐regulated DEGs *n* = 1650) (Figure 4c–e; Table S13). The quantity distribution exhibited a pattern similar to that observed for the DMGs, that is CR vs TM > CE versus TM > CR versus CE.

To elucidate the biological functions of the DEGs, we conducted GO and KEGG enrichment analyses (Figure S13a–c; Table S14). For GO analysis, three GO:BP categories associated with pathogen infection were enriched in the CR versus CE group, including defense response to virus (GO:0051607), defense response to symbiont (GO:0140546), and response to virus (GO:0009615). Several categories related to organismal responses to compounds were enriched in the CE versus TM group, including cellular response to toxic substances (GO:0097237) and response to oxygen‐containing compounds (GO:1901700). KEGG analysis indicated that the CR versus TM group was enriched in pathways previously associated with DMGs, particularly growth hormone synthesis, secretion, and action (KEGG:04935). Notably, DEGs in the CE versus TM group were significantly enriched in serotonergic synapse (KEGG:04726) and platelet activation (KEGG:04611), along with pathways linked to cancer and disease (Figure S14a–c; Table S15).

Among the three groups, we identified 339 shared DEGs (Figure S12). Based on further GO and KEGG enrichment analysis (Tables S14 and S15), these DEGs were enriched in inclusion body (GO:0016234) and porphyrin metabolism (KEGG:00860). Porphyrin metabolism is recognized as a vital process involved in heme synthesis (Bonkovsky et al., 2013).

### 2.4 Combined methylation and transcriptome analysis

**FIGURE 5:**
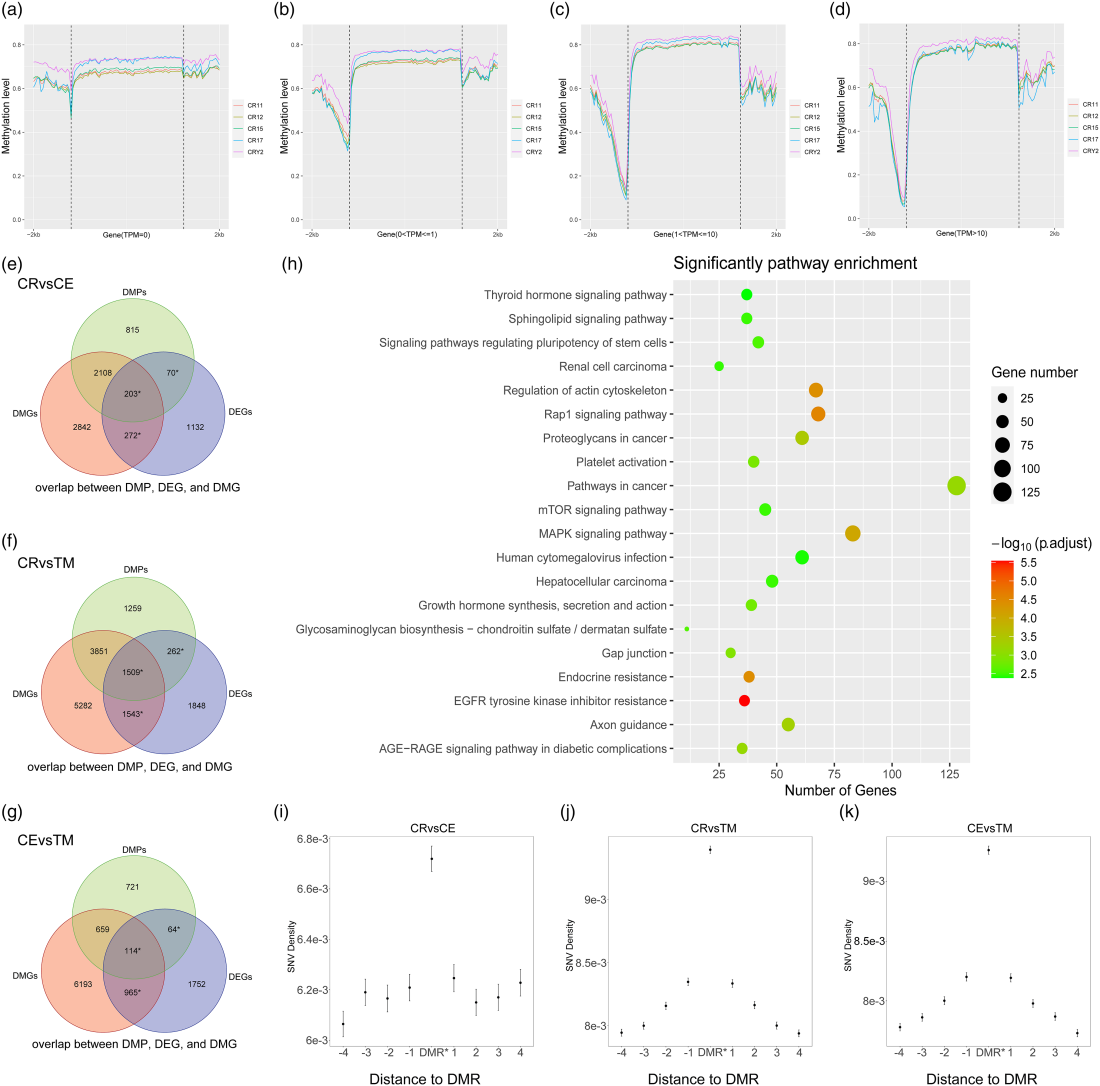
Combined analysis of multi‐omics. (a‐d) Methylation trends of 4 groups of CR genes with different expression intensities in the gene body and flanking regions. The corresponding expression intensity groups from (a) to (d) were TPM = 0, 0 < TPM ≤1, 1 < TPM ≤10, and TPM ≥10, respectively. (e–g) The multi‐omics differential gene. of (e) CR versus CE, (f) CR versus TM, and (g) CE versus TM. Part of the genes marked with asterisks were DMR‐related DEGs. (h) KEGG enrichment results of DMR‐related DEGs of CR versus TM. (i–k) SNV density of DMR and its flanking regions. In the figure, the abscissa origin DMR* represents DMR and ± 1 kb flanking sequence. ±1 ~ ±4 on the abscissa represents the 1 kb region from DMR ± 1 ~ ± 4 kb. The different SNV density groups from (i) to (k) are the hypermethylated DMGs of CR versus CE, CR versus TM, and CE versus TM.

To further explore the methylation of genes with different expression intensities near transcription start sites, we grouped the genes according to transcripts per million (TPM) intensity (TPM = 0, 0 < TPM ≤1, 1 < TPM ≤10, and TPM ≥10) to obtain methylation trends. From the perspective of gene expression intensity, methylation levels in the gene body and flanking regions changed with increasing TPM (Figure 5a–d; Figure S15a–h). In general, gene expression intensity increased with the decreasing methylation level in the 2‐kb region upstream of the gene body. In contrast, gene expression intensity increased slowly with methylation level of the gene body. Regions near the transcription start sites were associated with gene expression intensity and methylation levels (Jones, 2012). The methylation level in the 2‐kb region downstream of the gene body fluctuated around 0.7 with no apparent regularity. In the gene body region, we compared differences in methylation levels between different species under the same expression intensity (Figure 5a–d; Figure S15a–h). Results showed that the methylation level near the transcription start site decreased with increasing gene expression intensity, with the greatest decrease in CR, followed by CE and TM. These results suggest potential differences between species in how DNA methylation regulates gene expression.

To explore the association between the methylome and transcriptome, we conducted multi‐omics and enrichment analyses. We defined DEGs with DMRs on the promoter or gene body as DMR‐related DEGs (Table S16). According to analysis, the CR versus CE, CR versus TM, and CE versus TM groups contained 545, 3314, and 1143 DMR‐related DEGs, respectively (Figure 5e–g). Enrichment analysis was performed as above (Figure S16a–c; Tables S17 and S18). Due to the limited number of genes, the DMR‐related DEGs in the CR versus CE group showed no GO and KEGG enrichment. However, the DMR‐related DEGs in the CR vs TM group were enriched in several GO:MF categories, including protein kinase activity (GO:0004672) (Figure S16a) and KEGG pathways related to phenotypic differences, including growth hormone synthesis, secretion, and action (KEGG:04935) (Figure 5h). These results suggest that the phenotypic differences corresponding to KEGG pathways may be related to variation in gene expression levels caused by methylation differences. The DMR‐related DEGs in the CE versus TM group were enriched in several GO:CC categories (Figure S16b), and KEGG pathways (e.g., platelet activation (KEGG:04611)) (Figure S16c) also enriched in the CR versus TM group (Figure 5h).

### 2.5 Genomic variation of DMRs

Previous research has indicated that human hypermethylated DMRs exhibit more base mutations than other nearby regions (Hernando‐Herraez, Heyn, et al., 2015). To confirm the association between DMRs and base mutations among macaque species, we calculated the base mutation density within DMRs in different groups. Results revealed that hypermethylated DMRs displayed a higher base mutation density than their flanking regions, with methylation changes and an accelerated base mutation rate (Figure 5i–k). Conversely, the analysis showed a decrease in base mutation density in the CR versus CE and CR versus TM hypomethylated DMRs relative to the lateral region (Figure S17a,b), but an increase in base mutation density in the CE versus TM hypomethylated DMRs (Figure S17c). These findings suggest potential genetic conservation of base mutations in these regions. Moreover, both hypermethylated and hypomethylated DMRs displayed distinct base mutation patterns in the flanking sequence.

## 3 DISCUSSION

Despite the close relationship among macaque species, variations in their phenotypes, such as body size, exist. Whole‐genome sequencing studies have shed light on the evolutionary history and genetic distinctions among macaque species (Fan et al., 2014; Higashino et al., 2012; Osada et al., 2015; Yan et al., 2011). In our prior genomic study, we observed similarities in the genetic backgrounds of macaque species but also identified significant differences in specific genes, such as immune response genes and insulin‐related genes, among the different species (Fan et al., 2014). However, genetic variations at the DNA level alone cannot fully account for the observed variations in phenotypes among macaque species. Previous epigenetic studies on macaques have been limited, focusing on either a fraction of the genome or only on one species (e.g., rhesus macaques) without integrating genome and transcriptome data (Hata et al., 2013; Howard et al., 2011; Massart et al., 2014; Provençal et al., 2012). Here, we employed WGBS to obtain single‐base resolution methylation profiles of three macaque species and applied multi‐omics analysis of related genes affecting phenotypic differences. This study provides one of the first genome‐wide comparisons of genetic, gene expression, and epigenetic variation among different macaque species.

### 3.1 Genetic differences and DNA methylation profiles

Macaques are the most widely distributed nonhuman primates and serve as valuable animal models in biomedical research (Gibbs et al., 2007). Previous studies have reported the whole‐genome sequences of *M. mulatta lasiota*, *M. fascicularis*, and *M. thibetana* (Fan et al., 2014; Fang et al., 2011; Osada et al., 2015), revealing high similarity at the DNA level (with genome divergences around 0.28%–0.40%) (Fan et al., 2014; Osada et al., 2008; Osada et al., 2010). Despite the close genotypic relationship between CR and TM, which diverged approximately three million years ago, they also exhibit significant phenotypic differences (Fan et al., 2014; Perelman et al., 2011).

Various studies have investigated distinctions among macaque species from different perspectives. For instance, Li et al. (2020) used copy number variation (CNV) to construct a neighbor‐joining tree of nine *Macaca* species. Song et al. (2021) revealed that complex introgressive gene flow played an important role in the evolutionary history of *Macaca* and suggested that macaque evolution can be more accurately understood as phylogenetic networks rather than trees. Cui et al. (2019) reported similarities in the gut microbiota of CR and CE. They also found significantly higher alpha diversity in the rectal microbiota of CE compared to CR, despite a similar number of operational taxonomic units (OTUs), as well as significantly different relative abundances of dominant taxa, despite similar taxa at both the phylum and genus levels (Cui et al., 2019).

Fan et al. (2018) used whole‐genome sequences of several *Macaca* species to generate and analyze single nucleotide variant (SNV) data and found that the genetic distance between TM and CE was closer than that between TM and CR, with CR and CE exhibiting the closest genetic relationship. Our study revealed a relationship between DNA methylation patterns, differential gene expression, and species‐relatedness. Specifically, the greater the genetic distance between species, the more pronounced the differences in methylation and gene expression profiles. Similarly, we found that the methylation difference between CR and CE was the smallest, while that between CR and TM was the largest. Moreover, the number of DEGs in the CR versus CE group was the lowest, whereas that in the CR versus TM group was the highest. In addition, the distribution of DMR density in the CR versus CE and CR versus TM groups was more similar, indicating that methylation differences between CR and other species may occur at specific locations. These findings provide evidence of the correlation of the genome with the methylation profile, and the methylation profiles with the transcriptome.

Species have developed distinct adaptation patterns spanning from the genome to methylation groups and transcriptomes to adapt to different environments (Wong et al., 2005). The three macaque species studied here exhibit variations in their habitats and behaviors (Fleagle, 2013), and their adaptability to the environment could not solely be determined by genetics but also influenced by DNA methylation. Our functional enrichment analyses revealed that the DMR‐related genes (DMGs and DMPs) were enriched in developmental processes and several signaling pathways, including insulin secretion, thyroid hormone, morphine addiction, and the GABAergic synapse (Tables S10 and S12). Notably, the Wnt signaling pathway was enriched in multiple analyses. The Wnt signaling pathway plays a vital role in early embryonic development, organogenesis, tissue regeneration, and other physiological processes (Arkell et al., 2013). These patterns suggest that the DMRs are located in genomic regions involved in brain development and other traits, such as energy metabolism, which are particularly relevant to recent evolutionary history, as epigenetic involvement in human and primate brain evolution has been demonstrated (Mendizabal et al., 2016).

### 3.2 DMRs in metabolism‐related pathways

Considerable phenotypic differences, including variations in body size, exist among macaque species. TM exhibits the largest body size within the genus *Macaca*, while CE macaques are notably smaller than CR macaques. Macaque body size is influenced by several factors, such as social status and foraging behavior at an ethological level (Saito, 1996; Shively et al., 1997; Shively & Wallace, 2001), as well as hypothalamic‐pituitary‐adrenal axis function at an endocrine level (Kaufman et al., 2007). The growth and development of organisms are influenced by the endocrine signaling system in conjunction with signal transduction pathways (Neave, 2008). However, the specific impact of different levels of methylation on body size in the three macaque species has not yet been revealed. In our analysis of differential methylation among macaques, we identified several DMGs and DMPs that were enriched in pathways related to body size, growth, and development, including growth hormone synthesis, secretion, and action (KEGG:04935), insulin signaling pathway (KEGG:04910), thyroid hormone signaling pathway (KEGG:04919), and thyroid hormone synthesis (KEGG:04918) (Tables S10 and S12).

Notably, the growth hormone synthesis, secretion, and action pathway (KEGG:04935) was enriched in all DMG groups (Table S10). Among the identified DMGs, *SOCS1* is involved in the cell growth and cytoskeletal recombination stages of the growth hormone synthesis, secretion, and action pathway (Keren et al., 2006; Krebs & Hilton, 2001). Overexpression of *SOCS1* can completely inactivate the growth hormone (GH) signaling pathway (Ram & Waxman, 1999). Furthermore, *SOCS1* can inhibit GH receptor‐dependent JAK2 tyrosine phosphorylation and decrease GH activity (Ram & Waxman, 1999). Methylation of *SOCS1* can eliminate the inhibition of the JAK/STAT pathway at the methylation level, whereas demethylation of *SOCS1* can suppress cell growth and promote anchorage‐independent growth by inducing apoptosis (Yoshikawa et al., 2001). Therefore, we hypothesize that the up‐regulation of *SOCS1* gene expression in CR and CE compared to TM may be associated with the larger TM body size. DNA methylation can affect gene transcription near the transcription start site or in the gene body region. Therefore, the down‐regulation of *SOCS1* gene expression in TM may be attributed to hypermethylation. As a key downstream gene of the growth hormone pathway, *SOCS1* plays a crucial role in the growth and development of organisms. Hence, the down‐regulation of *SOCS1* gene expression in TM may be associated with its relatively large body size.

Blood glucose level is an essential factor affecting animal growth and development. Glucose‐induced insulin secretion and its potentiation constitute the principal mechanism of insulin release, while glucose metabolism generates adenosine triphosphate (ATP) to support daily activities (Seino et al., 2010). Studies have reported significant differences in blood glucose between CR and TM, with TM exhibiting relatively higher blood sugar levels (Yang et al., 2010). Fan et al. (2014) reported that Tibetan macaques contain more nonsynonymous mutations in genes involved in glucose metabolism, insulin secretion, and insulin receptor signaling pathways than rhesus and crab‐eating macaques. However, genomic differences alone may not fully explain the variations in genes related to insulin pathways, necessitating the examination of epigenetic differences. Our analysis revealed enrichment in the insulin signaling pathway (KEGG:04910) and insulin secretion (KEGG:04911) in all comparison groups (Tables S10 and S12), as well as enrichment in insulin receptors (*INSR*; *ENSMMUG00000028907*), which are essential components of insulin‐related pathways (Tables S10 and S12). In mammals, changes in insulin receptor levels are of physiological importance (Payankaulam et al., 2019). In animals, the physiological function of *INSR* is closely related to tyrosine kinase signal transduction (Ward et al., 2007). Heterozygous *INSR* mutant mice generally exhibit normal growth and fertility but show deficiencies in downstream signaling (Batista et al., 2019). In humans, heterozygous carriers of null mutations of *INSR* exhibit abnormal glucose tolerance, highlighting the importance of *INSR* gene expression (Psiachou et al., 1993). We identified *INSR* as a DMR‐related DEG in the CR versus CE and CR versus TM groups, with CR showing lower *INSR* gene expression compared to CE and TM. Therefore, both genomic and epigenetic differences were detected in identical/similar pathways, suggesting that these pathways may play crucial roles in the variation of blood glucose levels among macaque species.

Our results further showed the enrichment of DMGs in the thyroid hormone signaling pathway (KEGG:04919) in all comparison groups and thyroid hormone synthesis (KEGG:04918) in CR versus CE, CR versus TM, and the intersection of DMG groups (Tables S10and S12), which are essential in the regulation of growth, development, and metabolism (Cheng et al., 2010). KEGG analysis identified 58 (CR vs. CE), 95 (CR vs. TM), and 59 (CE vs. TM) DMGs enriched in the thyroid hormone signaling pathway (KEGG:04919). Interestingly, seven DMGs in the three groups (*ADCY1*, *ADCY2*, *ADCY3*, *ADCY5*, *ADCY7*, *ADCY8*, and *ADCY9*) were enriched in the same enzyme, adenylate cyclase (AC: EC 4.6.1.1), with *ADCY5* and *ADCY6* also identified as DMR‐related DEGs in the CR versus TM group. The enzyme protein kinase C (PKC: EC 2.7.11.13), related to *PRKCA* (ENSMMUG00000018378) and *PRKCB* (ENSMMUG00000014271), was found in all DMG groups. Furthermore, *PRKCA* was identified as a DMR‐related DEG in the CR versus TM group. We found that a certain number of thyroid hormone pathway‐related genes were differentially methylated, with some also showing differential expression. As the thyroid hormone‐related pathways regulate physiological functions, we consider that differential methylation may lead to corresponding physiological differences in the three macaque species.

### 3.3 DMRs in the neurosystem‐related pathway

In total, 34 (CR vs. CE), 69 (CR vs. TM), and 51 (CE vs. TM) DMGs were enriched in the GABAergic synapse pathway (KEGG:04727) (Tables S10 and S12). Gamma‐aminobutyric acid (GABA) is the most abundant inhibitory neurotransmitter in the mammalian central nervous system and binds to three major classes of receptors, that is, GABAA, GABAB, and GABAC (Chalifoux & Carter, 2011; Pinard et al., 2010). GABAA and GABAC receptors are ionotropic and mediate rapid GABA responses by triggering chloride channel opening, while GABAB receptors are metabotropic and mediate slower GABA responses by activating G‐proteins and influencing second messenger systems. We found that DMGs enriched in the GABAergic synapse pathway were related to protein subunits in the GABAA and GABAB receptors. Furthermore, *GABBR2* was identified as a DMG in the CR versus CE, CR vs TM, and CE versus TM groups. At the transcriptome level, *GABBR2* gene expression was down‐regulated in CR and CE compared with TM. In addition, the GABAA receptor‐related *GABRA2* gene (ENSMMUG00000003614) was identified as a DMR‐related DEG in the CR versus TM group.

The morphine addiction pathway (KEGG:05032) is closely associated with the GABAergic synapse pathway as morphine can indirectly excite ventral tegmental area dopamine neurons by reducing inhibitory synaptic transmission mediated by GABAergic neurons (Yan et al., 2008). Here, we identified 44 (CR vs. CE), 70 (CR vs. TM), and 64 (CE vs. TM) DMGs enriched in this pathway.

Our findings revealed differential methylation and gene expression patterns in neurosystem‐related genes among the three *Macaca* species, suggesting potential neurological differences among them. The observed variations in methylation and gene expression in neurosystem‐related genes may contribute to the differences in body size observed among the *Macaca* species due to endocrine regulation.

## 4 CONCLUSIONS

This study represents the first investigation into the genome‐wide DNA methylation patterns of rhesus, Tibetan, and crab‐eating macaques, providing valuable insights into their epigenetics and evolutionary biology. Furthermore, the transcriptomic analysis allowed us to investigate the relationship between differential methylation and gene expression. The enrichment of DMRs in pathways related to the nervous system and hormone regulation suggests an influence on energy metabolism and daily activities, as well as differentiation among species at the epigenetic level, which may partially explain the differences in body size and evolutionary history among macaques. However, our study only focuses on the differences between species, and the impact of other important factors, such as sex and age, on epigenetic inheritance also needs more in‐depth research. Besides, it is important to note that our study was limited to whole blood, and future research encompassing a wider range of tissues and samples within the genus *Macaca* will be necessary to comprehensively elucidate epigenomic differences. Including more species and samples in future studies will enhance our understanding of epigenetic variations among macaques.

## 5 MATERIALS AND METHODS

### 5.1 Samples of BS‐seq

Peripheral blood samples were collected from *M. mulatta lasiota* (CR; age: 1.5–18; male *n* = 3; female *n* = 2), *M. fascicularis* (CE; age: 2.3–11; male *n* = 2; female *n* = 3), and *M. thibetana* (TM; age: 3–15; male *n* = 3; female *n* = 2). Specific sample information is provided in the Supplementary Materials. We used whole blood from all species to represent general epigenomes of peripheral blood tissues (Hernando‐Herraez et al., 2013; Reinius et al., 2012). Interspecies DNA methylation variation may also result from genetic differences and/or environmental factors and other stochastic events (Hernando‐Herraez, Heyn, et al., 2015). Therefore, we used captive samples from all species to ensure homogenous environments.

### 5.2 BS‐seq data processing and analysis

Quality control and adapter trimming were performed using the NGSQCToolkit_v2.3 (Patel & Jain, 2012). Bismark was used to map the sequences after quality control with the reference genome (*M. mulatta*, Mmul_10), using the following parameters: ‐N 1‐L 20 ‐‐ Score_min L,0, −0.2 (Krueger & Andrews, 2011; Zhou et al., 2020). To obtain sample methylation coverage, we extracted methylation information from previously generated BAM files using Bismark (Krueger & Andrews, 2011). As DNA methylation primarily occurs in CpG dinucleotides in most animals, we only studied methylation at the CpG sites. To avoid the influence of sex differences and mitochondrial genomes, we removed methylation information from the X, Y, and MT chromosomes in subsequent analysis.

### 5.3 DNA methylation landscape

To explore the distribution pattern of DNA methylation in the three species, we used the R package ViewBS to analyze the methylation information for each sample (Huang et al., 2018). We used the R package circlize to determine methylation distribution in the genome (Gu et al., 2014). The methylation‐level distribution map was generated using a window size of 50 kb, while the DMR density statistics were calculated using a window size of 1e7 bp. The CpG sites with a methylation coverage ≥5 in the samples were extracted, and PCA and cluster analysis were performed using the R package methylKit, filters covering less than 10% of bases and more than 99.9% of bases, and a PCA plot with 95% confidence ellipses (Akalin et al., 2012).

### 5.4 Identification and analysis of DMRs

To identify DMRs between species, we analyzed CpG methylation site information using the R package DSS (Feng et al., 2014). The DMR extraction parameters were δ > 0.1 and *p* < 0.01, and the other parameters were the default values: minlen = 50, minCpG = 3, dis.merge = 50, pct.sig = 0.5. The obtained DMR information was compared with the information of various components of the reference genome using the R package GenomicRanges to search for overlapping areas (Lawrence et al., 2013). The gene annotation files were downloaded from the Ensembl website. The CpG_island information files were downloaded from the UCSC database. Promoter regions were defined as the sequence from 2‐kb before the transcription start site to the transcription start site. We used the online website g:Profiler to convert the ENST ID of the promoter into the ENSG ID of the corresponding gene (Raudvere et al., 2019). Finally, we obtained coverage information between DMRs and different components (UTR3, UTR5, promoter, gene body, exon, intron, CpG_island, and CpG_island‐related areas). Gene body regions with DMR coverage were defined as DMGs, while promoter regions with DMR coverage were defined as DMPs. To analyze the relationship between DMRs and transposable elements (TEs), we utilized RepeatMasker software to identify the TE regions (Smit, 2004). RepeatMasker uses default parameters to analyze the reference genome (Smit, 2004). We then searched for overlaps between TEs and DMRs. Enrichment analysis was also performed to explore the biological functions of DMR‐related genes. KEGG analysis was performed using g:Profiler with FDR <0.05 (Raudvere et al., 2019), while GO analysis was performed using the R package clusterProfiler with FDR <0.05 (Wu et al., 2021).

We also identified DMRs between male and female individuals using DSS same as the above method. Furthermore, we used the age‐related DMRs in rhesus macaques from our previous research (Zhou et al., 2020). Then, to indicate the effects of the age and sex to the species‐related DMRs, we identified the overlap DMRs between species‐related and age‐related, and between species‐related and sex‐related DMRs, using bedtools v2.25.0 (Quinlan & Hall, 2010).

### 5.5 Gene expression analysis

Another group of macaques, sequenced differently from the methylation group, was used for gene expression studies. Raw counts and TPM values of *M. thibetana* (TM; age: 15–18; male *n* = 2; female *n* = 3) and *M. mulatta lasiota* (CR; age: 15; male *n* = 3; female *n* = 3) were obtained from published laboratory studies (Wang et al., 2021). Transcriptome data of *M. fascicularis* (CE; age: 15–17; male *n* = 4; female *n* = 6) were sequenced and analyzed using the same procedures as previous studies (Wang et al., 2021).

For each sample with strict standards (high‐quality paired‐end reads with more than 90% of bases with Q‐value ≥30), we used NGSQCToolkit v2.3.3 to obtain high‐quality reads (clean reads) and remove low‐quality paired‐end reads or reads containing adaptors (Patel & Jain, 2012). The processed *M. fascicularis* reads were mapped to the reference genome (*M. mulatta*, Mmul_10) using HISAT2 v2.1.0 (Kim et al., 2015). Each alignment output file was assembled into a separate transcriptome using StringTie v1.3.6 to generate a transcriptome GTF file (Pertea et al., 2015). The genome sequence and annotations were downloaded from Ensembl release‐98. For *M. fascicularis*, specific Ensembl GTF files (Mmul_10.98) were used as the reference annotation file to guide the assembly process to obtain the TPM expression value and raw read counts for each gene and transcript.

We used the generalized linear model (GLM) in the R package DESeq2 (Fitting formula: ~species + sex) to analyze pairwise differential expression in transcriptome data of the three species (Love et al., 2014). The Wald test was used for pairwise comparisons of the differentially expressed transcriptomes, and the GLM was fit to estimate size factors and dispersity of all samples from each species. The significance threshold of DEGs was set as adjusted *p* < 0.05 and abs (log2Fold‐Change) > 1. To eliminate interference, we excluded genes with count values <10 and loci that were not autosomal. The obtained gene expression information was used for subsequent correlation analysis.

### 5.6 Analysis of genetic differences in DMRs

We analyzed the base mutation information in the DMRs and flanking regions to investigate the relationship between genomic differences and DNA methylation. Genomic data for the three species were obtained from unpublished laboratory work. Previous studies excluded insertions and deletions from the genome when calculating base differences between pairs of species (Hernando‐Herraez, Heyn, et al., 2015).

## FUNDING INFORMATION

This work was supported by the National Natural Science Foundation of China (32070413).

## CONFLICT OF INTEREST STATEMENT

The authors declare that they have no competing interests.

## Supporting information


Appendix S1.

Appendix S2.

Appendix S3.

## Data Availability

Clean data of BS‐seq and RNA‐seq have been submitted to China National GenBank (CNGB) with the project accession CNP0002974.

## References

[eva13604-bib-0001] Akalin, A. , Kormaksson, M. , Li, S. , Garrett‐Bakelman, F. E. , Figueroa, M. E. , Melnick, A. , & Mason, C. E. (2012). methylKit: A comprehensive R package for the analysis of genome‐wide DNA methylation profiles. Genome Biology, 13(10), R87. 23034086 10.1186/gb-2012-13-10-r87 PMC3491415

[eva13604-bib-0002] Angers, B. , Castonguay, E. , & Massicotte, R. (2010). Environmentally induced phenotypes and DNA methylation: How to deal with unpredictable conditions until the next generation and after. Molecular Ecology, 19(7), 1283–1295. 20298470 10.1111/j.1365-294X.2010.04580.x

[eva13604-bib-0003] Batista, T. M. , Garcia‐Martin, R. , Cai, W. , Konishi, M. , O’Neill, B. T. , Sakaguchi, M. , Kim, J. H. , Jung, D. Y. , Kim, J. K. , & Kahn, C. R. (2019). Multi‐dimensional transcriptional remodeling by physiological insulin in vivo. Cell Reports, 26(12), 3429–3443.e3423. 30893613 10.1016/j.celrep.2019.02.081 PMC6543850

[eva13604-bib-0004] Bonkovsky, H. L. , Guo, J. T. , Hou, W. , Li, T. , Narang, T. , & Thapar, M. (2013). Porphyrin and heme metabolism and the porphyrias. Comprehensive Physiology, 3(1), 365–401. 23720291 10.1002/cphy.c120006

[eva13604-bib-0005] Chalifoux, J. R. , & Carter, A. G. (2011). GABAB receptor modulation of synaptic function. Current Opinion in Neurobiology, 21(2), 339–344. 21376567 10.1016/j.conb.2011.02.004 PMC3092847

[eva13604-bib-0006] Cheng, S.‐Y. , Leonard, J. L. , & Davis, P. J. (2010). Molecular aspects of thyroid hormone actions. Endocrine Reviews, 31(2), 139–170. 20051527 10.1210/er.2009-0007 PMC2852208

[eva13604-bib-0007] Cui, Y.‐F. , Wang, F.‐J. , Yu, L. , Ye, H.‐H. , & Yang, G.‐B. (2019). Metagenomic comparison of the rectal microbiota between rhesus macaques (*Macaca mulatta*) and cynomolgus macaques (*Macaca fascicularis*). Zoological Research, 40(2), 89–93. 30127329 10.24272/j.issn.2095-8137.2018.061 PMC6378564

[eva13604-bib-0008] Fan, Z. , Zhao, G. , Li, P. , Osada, N. , Xing, J. , Yi, Y. , du, L. , Silva, P. , Wang, H. , Sakate, R. , Zhang, X. , Xu, H. , Yue, B. , & Li, J. (2014). Whole‐genome sequencing of tibetan macaque (*Macaca thibetana*) provides new insight into the macaque evolutionary history. Molecular Biology and Evolution, 31(6), 1475–1489. 24648498 10.1093/molbev/msu104 PMC4032132

[eva13604-bib-0009] Fan, Z. , Zhou, A. , Osada, N. , Yu, J. , Jiang, J. , Li, P. , du, L. , Niu, L. , Deng, J. , Xu, H. , Xing, J. , Yue, B. , & Li, J. (2018). Ancient hybridization and admixture in macaques (genus *Macaca*) inferred from whole genome sequences. Molecular Phylogenetics and Evolution, 127, 376–386. 29614345 10.1016/j.ympev.2018.03.038

[eva13604-bib-0010] Fang, X. , Zhang, Y. , Zhang, R. , Yang, L. , Li, M. , Ye, K. , Guo, X. , Wang, J. , & Su, B. (2011). Genome sequence and global sequence variation map with 5.5 million SNPs in Chinese rhesus macaque. Genome Biology, 12(7), R63. 21733155 10.1186/gb-2011-12-7-r63 PMC3218825

[eva13604-bib-0011] Feng, H. , Conneely, K. N. , & Wu, H. (2014). A Bayesian hierarchical model to detect differentially methylated loci from single nucleotide resolution sequencing data. Nucleic Acids Research, 42(8), e69. 24561809 10.1093/nar/gku154 PMC4005660

[eva13604-bib-0012] Fleagle, J. G. (2013). Chapter 6 ‐ Old World monkeys. In Fleagle JG. Primate adaptation and evolution (Third ed., pp. 119–150). Academic Press.

[eva13604-bib-0013] Fraga, M. F. , Ballestar, E. , Paz, M. F. , Ropero, S. , Setien, F. , Ballestar, M. L. , Heine‐Suñer, D. , Cigudosa, J. C. , Urioste, M. , Benitez, J. , Boix‐Chornet, M. , Sanchez‐Aguilera, A. , Ling, C. , Carlsson, E. , Poulsen, P. , Vaag, A. , Stephan, Z. , Spector, T. D. , Wu, Y. Z. , … Esteller, M. (2005). Epigenetic differences arise during the lifetime of monozygotic twins. Proceedings of the National Academy of Sciences of the United States of America, 102(30), 10604. 16009939 10.1073/pnas.0500398102 PMC1174919

[eva13604-bib-0014] Gibbs, R. A. , Rogers, J. , Katze, M. G. , Bumgarner, R. , Weinstock, G. M. , Mardis, E. R. , Remington, K. A. , Strausberg, R. L. , Venter, J. C. , Wilson, R. K. , Batzer, M. A. , Bustamante, C. D. , Eichler, E. E. , Hahn, M. W. , Hardison, R. C. , Makova, K. D. , Miller, W. , Milosavljevic, A. , Palermo, R. E. , … Zwieg, A. S. (2007). Evolutionary and biomedical insights from the rhesus macaque genome. Science, 316(5822), 222–234. 17431167 10.1126/science.1139247

[eva13604-bib-0015] Gu, Z. , Gu, L. , Eils, R. , Schlesner, M. , & Brors, B. (2014). Circlize implements and enhances circular visualization in R. Bioinformatics (Oxford, England), 30(19), 2811–2812. 24930139 10.1093/bioinformatics/btu393

[eva13604-bib-0016] Hata, K. , Mizukami, H. , Sadakane, O. , Watakabe, A. , Ohtsuka, M. , Takaji, M. , Kinoshita, M. , Isa, T. , Ozawa, K. , & Yamamori, T. (2013). DNA methylation and methyl‐binding proteins control differential gene expression in distinct cortical areas of macaque monkey. The Journal of Neuroscience: The Official Journal of the Society for Neuroscience, 33(50), 19704–19714. 24336734 10.1523/JNEUROSCI.2355-13.2013 PMC6618758

[eva13604-bib-0017] Hernando‐Herraez, I. , Garcia‐Perez, R. , Sharp, A. J. , & Marques‐Bonet, T. (2015). DNA methylation: Insights into human evolution. PLoS Genetics, 11(12), e1005661. 26658498 10.1371/journal.pgen.1005661 PMC4684328

[eva13604-bib-0018] Hernando‐Herraez, I. , Heyn, H. , Fernandez‐Callejo, M. , Vidal, E. , Fernandez‐Bellon, H. , Prado‐Martinez, J. , Sharp, A. J. , Esteller, M. , & Marques‐Bonet, T. (2015). The interplay between DNA methylation and sequence divergence in recent human evolution. Nucleic Acids Research, 43(17), 8204–8214. 26170231 10.1093/nar/gkv693 PMC4787803

[eva13604-bib-0019] Hernando‐Herraez, I. , Prado‐Martinez, J. , Garg, P. , Fernandez‐Callejo, M. , Heyn, H. , Hvilsom, C. , Navarro, A. , Esteller, M. , Sharp, A. J. , & Marques‐Bonet, T. (2013). Dynamics of DNA methylation in recent human and great ape evolution. PLoS Genetics, 9(9), e1003763. 24039605 10.1371/journal.pgen.1003763 PMC3764194

[eva13604-bib-0020] Heyn, H. , Moran, S. , Hernando‐Herraez, I. , Sayols, S. , Gomez, A. , Sandoval, J. , Monk, D. , Hata, K. , Marques‐Bonet, T. , Wang, L. , & Esteller, M. (2013). DNA methylation contributes to natural human variation. Genome Research, 23(9), 1363–1372. 23908385 10.1101/gr.154187.112 PMC3759714

[eva13604-bib-0021] Higashino, A. , Sakate, R. , Kameoka, Y. , Takahashi, I. , Hirata, M. , Tanuma, R. , Masui, T. , Yasutomi, Y. , & Osada, N. (2012). Whole‐genome sequencing and analysis of the Malaysian cynomolgus macaque (*Macaca fascicularis*) genome. Genome Biology, 13(7), R58. 22747675 10.1186/gb-2012-13-7-r58 PMC3491380

[eva13604-bib-0022] Hodges, E. , Molaro, A. , Dos Santos, C. O. , Thekkat, P. , Song, Q. , Uren, P. J. , Park, J. , Butler, J. , Rafii, S. , McCombie, W. R. , Smith, A. D. , & Hannon, G. J. (2011). Directional DNA methylation changes and complex intermediate states accompany lineage specificity in the adult hematopoietic compartment. Molecular Cell, 44(1), 17–28. 21924933 10.1016/j.molcel.2011.08.026 PMC3412369

[eva13604-bib-0023] Howard, T. D. , Ho, S.‐M. , Zhang, L. , Chen, J. , Cui, W. , Slager, R. , Gray, S. , Hawkins, G. A. , Medvedovic, M. , & Wagner, J. D. (2011). Epigenetic changes with dietary soy in cynomolgus monkeys. PLoS One, 6(10), e26791. 22046358 10.1371/journal.pone.0026791 PMC3201974

[eva13604-bib-0024] Huang, X. , Zhang, S. , Li, K. , Thimmapuram, J. , Xie, S. , & Wren, J. (2018). ViewBS: A powerful toolkit for visualization of high‐throughput bisulfite sequencing data. Bioinformatics (Oxford, England), 34(4), 708–709. 29087450 10.1093/bioinformatics/btx633 PMC5860610

[eva13604-bib-0025] Jeremias, G. , Barbosa, J. , Marques, S. M. , Asselman, J. , Gonçalves, F. J. M. , & Pereira, J. L. (2018). Synthesizing the role of epigenetics in the response and adaptation of species to climate change in freshwater ecosystems. Molecular Ecology, 27(13), 2790–2806. 29802778 10.1111/mec.14727

[eva13604-bib-0026] Jones, P. A. (2012). Functions of DNA methylation: Islands, start sites, gene bodies and beyond. Nature Reviews. Genetics, 13(7), 484–492. 10.1038/nrg3230 22641018

[eva13604-bib-0027] Kaminsky, Z. A. , Tang, T. , Wang, S.‐C. , Ptak, C. , Oh, G. H. T. , Wong, A. H. C. , Feldcamp, L. A. , Virtanen, C. , Halfvarson, J. , Tysk, C. , McRae, A. F. , Visscher, P. M. , Montgomery, G. W. , Gottesman, I. I. , Martin, N. G. , & Petronis, A. (2009). DNA methylation profiles in monozygotic and dizygotic twins. Nature Genetics, 41(2), 240–245. 19151718 10.1038/ng.286

[eva13604-bib-0028] Kaufman, D. , Banerji, M. A. , Shorman, I. , Smith, E. L. P. , Coplan, J. D. , Rosenblum, L. A. , & Kral, J. G. (2007). Early‐life stress and the development of obesity and insulin resistance in juvenile bonnet macaques. Diabetes, 56(5), 1382–1386. 17470564 10.2337/db06-1409

[eva13604-bib-0029] Keren, A. , Tamir, Y. , & Bengal, E. (2006). The p38 MAPK signaling pathway: A major regulator of skeletal muscle development. Molecular and Cellular Endocrinology, 252(1), 224–230. 16644098 10.1016/j.mce.2006.03.017

[eva13604-bib-0030] Kim, D. , Langmead, B. , & Salzberg, S. L. (2015). HISAT: A fast spliced aligner with low memory requirements. Nature Methods, 12(4), 357–360. 25751142 10.1038/nmeth.3317 PMC4655817

[eva13604-bib-0031] Klughammer, J. , Romanovskaia, D. , Nemc, A. , Posautz, A. , Seid, C. A. , Schuster, L. C. , Keinath, M. C. , Lugo Ramos, J. S. , Kosack, L. , Evankow, A. , Printz, D. , Kirchberger, S. , Ergüner, B. , Datlinger, P. , Fortelny, N. , Schmidl, C. , Farlik, M. , Skjærven, K. , Bergthaler, A. , … Bock, C. (2023). Comparative analysis of genome‐scale, base‐resolution DNA methylation profiles across 580 animal species. Nature Communications, 14(1), 232. 10.1038/s41467-022-34828-y PMC9842680 36646694

[eva13604-bib-0032] Krebs, D. L. , & Hilton, D. J. (2001). SOCS proteins: Negative regulators of cytokine signaling. Stem Cells, 19(5), 378–387. 11553846 10.1634/stemcells.19-5-378

[eva13604-bib-0033] Krueger, F. , & Andrews, S. R. (2011). Bismark: A flexible aligner and methylation caller for bisulfite‐seq applications. Bioinformatics (Oxford, England), 27(11), 1571–1572. 21493656 10.1093/bioinformatics/btr167 PMC3102221

[eva13604-bib-0034] Kucharski, R. , Maleszka, J. , Foret, S. , & Maleszka, R. (2008). Nutritional control of reproductive status in honeybees via DNA methylation. Science, 319(5871), 1827–1830. 18339900 10.1126/science.1153069

[eva13604-bib-0035] Lan, Y. , Wang, J. , Yang, Q. , Tang, R.‐X. , Zhou, M. , Lei, G.‐L. , Li, J. , Zhang, L. , Yue, B. S. , & Fan, Z. X. (2020). Blood transcriptome analysis reveals gene expression features of breast‐feeding rhesus macaque (*Macaca mulatta*) infants. Zoological Research, 41(4), 431–436. 32400975 10.24272/j.issn.2095-8137.2020.044 PMC7340523

[eva13604-bib-0036] Lawrence, M. , Huber, W. , Pagès, H. , Aboyoun, P. , Carlson, M. , Gentleman, R. , Morgan, M. T. , & Carey, V. J. (2013). Software for computing and annotating genomic ranges. PLoS Computational Biology, 9(8), e1003118. 23950696 10.1371/journal.pcbi.1003118 PMC3738458

[eva13604-bib-0037] Li, J. , Fan, Z. , Shen, F. , Pendleton, A. L. , Song, Y. , Xing, J. , Yue, B. , Kidd, J. M. , & Li, J. (2020). Genomic copy number variation study of nine *Macaca* species provides new insights into their genetic divergence, adaptation, and biomedical application. Genome Biology and Evolution, 12(12), 2211–2230. 32970804 10.1093/gbe/evaa200 PMC7846157

[eva13604-bib-0038] Love, M. I. , Huber, W. , & Anders, S. (2014). Moderated estimation of fold change and dispersion for RNA‐seq data with DESeq2. Genome Biology, 15(12), 550. 25516281 10.1186/s13059-014-0550-8 PMC4302049

[eva13604-bib-0039] Lyko, F. , Foret, S. , Kucharski, R. , Wolf, S. , Falckenhayn, C. , & Maleszka, R. (2010). The honey bee epigenomes: Differential methylation of brain DNA in Queens and workers. PLoS Biology, 8(11), e1000506. 21072239 10.1371/journal.pbio.1000506 PMC2970541

[eva13604-bib-0040] Massart, R. , Suderman, M. , Provencal, N. , Yi, C. , Bennett, A. J. , Suomi, S. , & Szyf, M. (2014). Hydroxymethylation and DNA methylation profiles in the prefrontal cortex of the non‐human primate rhesus macaque and the impact of maternal deprivation on hydroxymethylation. Neuroscience, 268, 139–148. 24657458 10.1016/j.neuroscience.2014.03.021 PMC6528794

[eva13604-bib-0041] Mendizabal, I. , Shi, L. , Keller, T. E. , Konopka, G. , Preuss, T. M. , Hsieh, T.‐F. , Hu, E. , Zhang, Z. , Su, B. , & Yi, S. V. (2016). Comparative methylome analyses identify epigenetic regulatory loci of human brain evolution. Molecular Biology and Evolution, 33(11), 2947–2959. 27563052 10.1093/molbev/msw176 PMC5062329

[eva13604-bib-0042] Molaro, A. , Hodges, E. , Fang, F. , Song, Q. , Mccombie, W. R. , Hannon, G. J. , & Smith, A. D. (2011). Sperm methylation profiles reveal features of epigenetic inheritance and evolution in primates. Cell, 146(6), 1029–1041. 21925323 10.1016/j.cell.2011.08.016 PMC3205962

[eva13604-bib-0043] Neave, N. (2008). Hormones and behaviour: A psychological approach. Cambridge University Press.

[eva13604-bib-0044] Osada, N. , Hashimoto, K. , Kameoka, Y. , Hirata, M. , Tanuma, R. , Uno, Y. , Inoue, I. , Hida, M. , Suzuki, Y. , Sugano, S. , Terao, K. , Kusuda, J. , & Takahashi, I. (2008). Large‐scale analysis of *Macaca fascicularis* transcripts and inference of genetic divergence between *M‐fascicularis* and *M‐mulatta* . BMC Genomics, 9, 90. 18294402 10.1186/1471-2164-9-90 PMC2287170

[eva13604-bib-0045] Osada, N. , Hettiarachchi, N. , Adeyemi Babarinde, I. , Saitou, N. , & Blancher, A. (2015). Whole‐genome sequencing of six Mauritian cynomolgus macaques (*Macaca fascicularis*) reveals a genome‐wide pattern of polymorphisms under extreme population bottleneck. Genome Biology and Evolution, 7(3), 821–830. 25805843 10.1093/gbe/evv033 PMC5322541

[eva13604-bib-0046] Osada, N. , Uno, Y. , Mineta, K. , Kameoka, Y. , Takahashi, I. , & Terao, K. (2010). Ancient genome‐wide admixture extends beyond the current hybrid zone between *Macaca fascicularis* and M‐mulatta. Molecular Ecology, 19(14), 2884–2895. 20579289 10.1111/j.1365-294X.2010.04687.x

[eva13604-bib-0047] Patel, R. K. , & Jain, M. (2012). NGS QC toolkit: A toolkit for quality control of next generation sequencing data. PLoS One, 7(2), e30619. 22312429 10.1371/journal.pone.0030619 PMC3270013

[eva13604-bib-0048] Payankaulam, S. , Raicu, A.‐M. , & Arnosti, D. N. (2019). Transcriptional regulation of INSR, the insulin receptor gene. Genes, 10(12), 984. 31795422 10.3390/genes10120984 PMC6947883

[eva13604-bib-0049] Perelman, P. , Johnson, W. E. , Roos, C. , Seuánez, H. N. , Horvath, J. E. , Moreira, M. A. , Kessing, B. , Pontius, J. , Roelke, M. , Rumpler, Y. , Schneider, M. P. , Silva, A. , O'Brien, S. J. , & Pecon‐Slattery, J. (2011). A molecular phylogeny of living primates. PLoS Genetics, 7(3), e1001342. 21436896 10.1371/journal.pgen.1001342 PMC3060065

[eva13604-bib-0050] Pertea, M. , Pertea, G. M. , Antonescu, C. M. , Chang, T.‐C. , Mendell, J. T. , & Salzberg, S. L. (2015). StringTie enables improved reconstruction of a transcriptome from RNA‐seq reads. Nature Biotechnology, 33(3), 290–295. 10.1038/nbt.3122 PMC4643835 25690850

[eva13604-bib-0051] Pinard, A. , Seddik, R. , & Bettler, B. (2010). GABAB receptors: Physiological functions and mechanisms of diversity. In Blackburn TP. Advances in pharmacology (pp. 231–255). Academic Press. 10.1016/S1054-3589(10)58010-4 20655485

[eva13604-bib-0052] Provençal, N. , Suderman, M. J. , Guillemin, C. , Massart, R. , Ruggiero, A. , Wang, D. , Bennett, A. J. , Pierre, P. J. , Friedman, D. P. , Côté, S. M. , Hallett, M. , Tremblay, R. E. , Suomi, S. J. , & Szyf, M. (2012). The signature of maternal rearing in the methylome in rhesus macaque prefrontal cortex and T cells. The Journal of Neuroscience: The Official Journal of the Society for Neuroscience, 32(44), 15626–15642. 23115197 10.1523/JNEUROSCI.1470-12.2012 PMC3490439

[eva13604-bib-0053] Psiachou, H. , Mitton, S. , Alaghband‐Zadeh, J. , Hone, J. , Taylor, S. , & Sinclair, L. (1993). Leprechaunism and homozygous nonsense mutation in the insulin receptor gene. The Lancet, 342(8876), 924. 10.1016/0140-6736(93)91970-w 8105179

[eva13604-bib-0054] Quinlan, A. R. , & Hall, I. M. (2010). BEDTools: A flexible suite of utilities for comparing genomic features. Bioinformatics, 26(6), 841–842. 20110278 10.1093/bioinformatics/btq033 PMC2832824

[eva13604-bib-0055] Ram, P. A. , & Waxman, D. J. (1999). SOCS/CIS protein inhibition of growth hormone‐stimulated STAT5 signaling by multiple mechanisms*. Journal of Biological Chemistry, 274(50), 35553–35561. 10585430 10.1074/jbc.274.50.35553

[eva13604-bib-0056] Raudvere, U. , Kolberg, L. , Kuzmin, I. , Arak, T. , Adler, P. , Peterson, H. , & Vilo, J. (2019). G:Profiler: A web server for functional enrichment analysis and conversions of gene lists (2019 update). Nucleic Acids Research, 47(W1), W191–W198. 31066453 10.1093/nar/gkz369 PMC6602461

[eva13604-bib-0057] Reinius, L. E. , Acevedo, N. , Joerink, M. , Pershagen, G. , Dahlén, S.‐E. , Greco, D. , Söderhäll, C. , Scheynius, A. , & Kere, J. (2012). Differential DNA methylation in purified human blood cells: Implications for cell lineage and studies on disease susceptibility. PLoS One, 7(7), e41361. 22848472 10.1371/journal.pone.0041361 PMC3405143

[eva13604-bib-0058] Saito, C. (1996). Dominance and feeding success in female Japanese macaques, *Macaca fuscata*: Effects of food patch size and inter‐patch distance. Animal Behaviour, 51(5), 967–980.

[eva13604-bib-0059] Seino, S. , Shibasaki, T. , & Minami, K. (2010). Pancreatic beta‐cell signaling: Toward better understanding of diabetes and its treatment. Proceedings of the Japan Academy. Series B, Physical and Biological Sciences, 86(6), 563–577. 20551594 10.2183/pjab.86.563 PMC3081169

[eva13604-bib-0060] Shively, C. A. , Laber‐Laird, K. , & Anton, R. F. (1997). Behavior and physiology of social stress and depression in female cynomolgus monkeys. Biological Psychiatry, 41(8), 871–882. 9099414 10.1016/S0006-3223(96)00185-0

[eva13604-bib-0061] Shively, C. A. , & Wallace, J. M. (2001). Social status, social stress and fat distribution in primates. In International textbook of obesity (pp. 203–211). Wiley.

[eva13604-bib-0062] Smit, A. F. A. (2004). Repeat‐Masker Open‐3.0. http://www.repeatmasker.org

[eva13604-bib-0063] Solari, S. , & Baker, R. J. (2007). Mammal species of the world: A taxonomic and geographic reference by D. E. Wilson; D. M. Reeder. Journal of Mammalogy, 88(3), 824–830.

[eva13604-bib-0064] Song, Y. , Jiang, C. , Li, K.‐H. , Li, J. , Qiu, H. , Price, M. , Fan, Z. X. , Li, J. (2021). Genome‐wide analysis reveals signatures of complex introgressive gene flow in macaques (genus *Macaca*). Zoological Research, 42(4), 433–449. 34114757 10.24272/j.issn.2095-8137.2021.038 PMC8317189

[eva13604-bib-0065] Wang, J. , Lan, Y. , He, L. , Tang, R. , Li, Y. , Huang, Y. , Liang, S. , Gao, Z. , Price, M. , Yue, B. , He, M. , Guo, T. , & Fan, Z. (2021). Sex‐specific gene expression in the blood of four primates. Genomics, 113(4), 2605–2613. 34116169 10.1016/j.ygeno.2021.06.007

[eva13604-bib-0066] Ward, C. W. , Lawrence, M. C. , Streltsov, V. A. , Adams, T. E. , & Mckern, N. M. (2007). The insulin and EGF receptor structures: New insights into ligand‐induced receptor activation. Trends in Biochemical Sciences, 32(3), 129–137. 17280834 10.1016/j.tibs.2007.01.001

[eva13604-bib-0067] Warren Wesley, C. , Harris, R. A. , Haukness, M. , Fiddes Ian, T. , Murali Shwetha, C. , Fernandes, J. , Dishuck, P. C. , Storer, J. M. , Raveendran, M. , Hillier, L. W. , Porubsky, D. , Mao, Y. , Gordon, D. , Vollger, M. R. , Lewis, A. P. , Munson, K. M. , DeVogelaere, E. , Armstrong, J. , Diekhans, M. , … Eichler, E. E. (2020). Sequence diversity analyses of an improved rhesus macaque genome enhance its biomedical utility. Science, 370(6523), eabc6617. 33335035 10.1126/science.abc6617 PMC7818670

[eva13604-bib-0068] Wong, A. H. C. , Gottesman, I. I. , & Petronis, A. (2005). Phenotypic differences in genetically identical organisms: The epigenetic perspective. Human Molecular Genetics, 14(suppl_1), R11–R18. 15809262 10.1093/hmg/ddi116

[eva13604-bib-0069] Wu, T. , Hu, E. , Xu, S. , Chen, M. , Guo, P. , Dai, Z. , Feng, T. , Zhou, L. , Tang, W. , Zhan, L. , Fu, X. , Liu, S. , Bo, X. , & Yu, G. (2021). clusterProfiler 4.0: A universal enrichment tool for interpreting omics data. Innovation (New York, N.Y.), 2(3), 100141. 10.1016/j.xinn.2021.100141 PMC8454663 34557778

[eva13604-bib-0070] Xu, J. , Lan, Y. , Wang, X. , Shang, K. , Liu, X. , Wang, J. , Li, J. , Yue, B. , Shao, M. , & Fan, Z. (2022). Multi‐omics analysis reveals the host‐microbe interactions in aged rhesus macaques. Frontiers in Microbiology, 13, 993879. 36238598 10.3389/fmicb.2022.993879 PMC9551614

[eva13604-bib-0071] Yan, C.‐C. , Zhang, X.‐S. , Zhou, L. , Yang, Q. , Zhou, M. , Zhang, L.‐W. , Xing, J. C. , Yan, Z. F. , Price, M. , Li, J. , Yue, B. S. , Fan, Z. X. (2020). Effects of aging on gene expression in blood of captive Tibetan macaques (*Macaca thibetana*) and comparisons with expression in humans. Zoological Research, 41(5), 557–563. 32746507 10.24272/j.issn.2095-8137.2020.092 PMC7475009

[eva13604-bib-0072] Yan, G. , Zhang, G. , Fang, X. , Zhang, Y. , Li, C. , Ling, F. , Cooper, D. N. , Li, Q. , Li, Y. , van Gool, A. J. , du, H. , Chen, J. , Chen, R. , Zhang, P. , Huang, Z. , Thompson, J. R. , Meng, Y. , Bai, Y. , Wang, J. , … Wang, J. (2011). Genome sequencing and comparison of two nonhuman primate animal models, the cynomolgus and Chinese rhesus macaques. Nature Biotechnology, 29(11), 1019–1023. 10.1038/nbt.1992 22002653

[eva13604-bib-0073] Yan, Z. , Qiuyue, C. , & Yu, L.‐C. (2008). Morphine: A protective or destructive role in neurons? The Neuroscientist, 14(6), 561–570. 18349442 10.1177/1073858408314434

[eva13604-bib-0074] Yang, F. , Wang, H. X. , Zhou, L. , Ai, Y. X. , & Zeng, T. (2010). A primary analyze and measurement on partial biochemistry index of peripheral blood cells of *Macaca thibetana* . Sichuan Journal of Zoology, 29, 256–258.

[eva13604-bib-0075] Yoshikawa, H. , Matsubara, K. , Qian, G.‐S. , Jackson, P. , Groopman, J. D. , Manning, J. E. , Harris, C. C. , & Herman, J. G. (2001). SOCS‐1, a negative regulator of the JAK/STAT pathway, is silenced by methylation in human hepatocellular carcinoma and shows growth‐suppression activity. Nature Genetics, 28(1), 29–35. 11326271 10.1038/ng0501-29

[eva13604-bib-0076] Zhou, M. , Zhang, L. , Yang, Q. , Yan, C. , Jiang, P. , Lan, Y. , Wang, J. , Tang, R. , He, M. , Lei, G. , Sun, P. , Su, N. , Price, M. , Li, J. , Lin, F. , Yue, B. , & Fan, Z. (2020). Age‐related gene expression and DNA methylation changes in rhesus macaque. Genomics, 112(6), 5147–5156. 32927008 10.1016/j.ygeno.2020.09.021

