# Supplementary material for: DNA methylation and transcriptome analysis reveal epigenomic differences among three macaque species
Source: Evol Appl. 2023 Oct 12;17(2):e13604. doi: 10.1111/eva.13604 (PMC10853583; doi:10.1111/eva.13604)
Supplement: Supplementary file 1 — Appendix S1. [file EVA-17-e13604-s002.pdf]

Supplemental figures

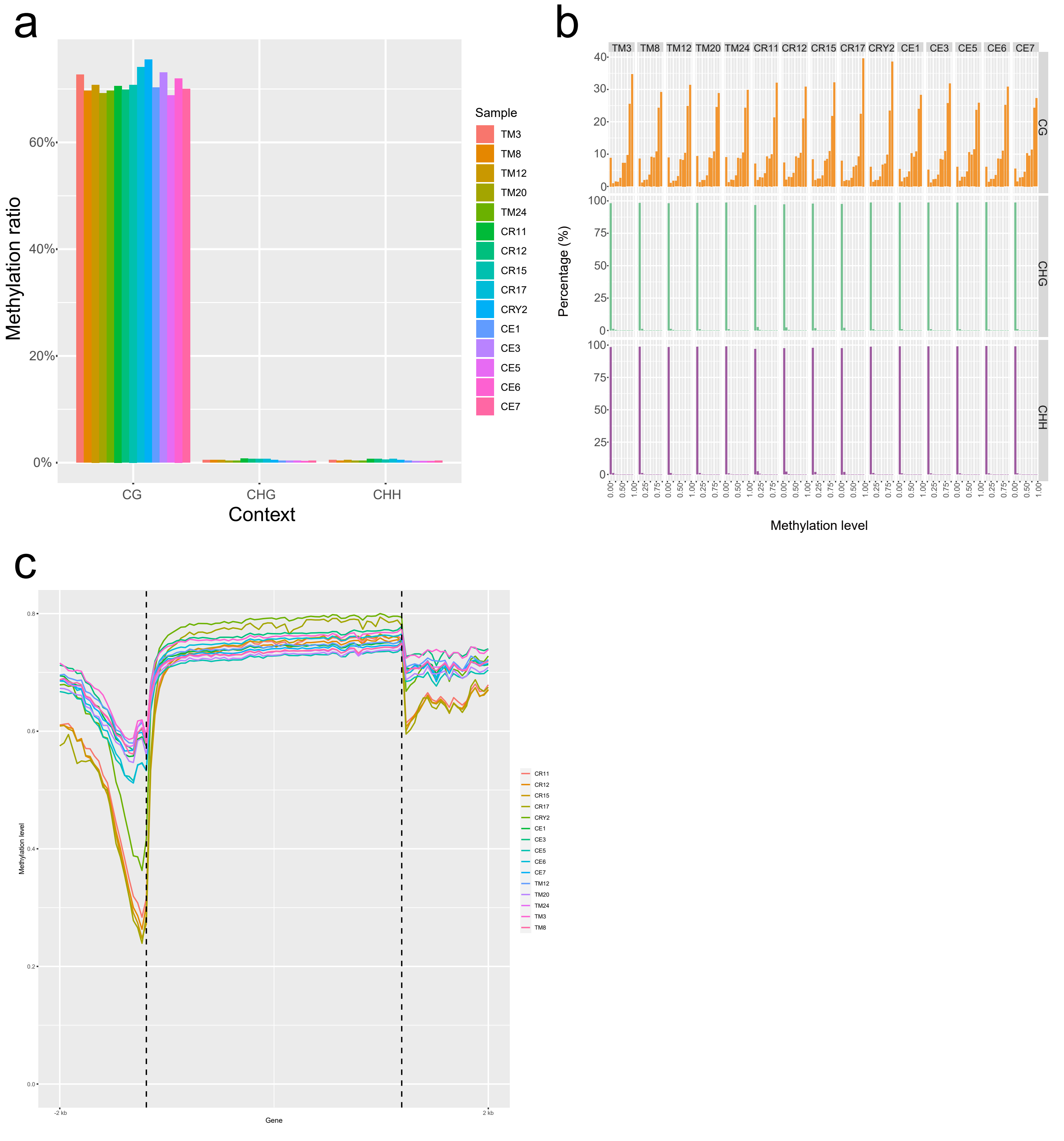

Supplementary Fig. S1 (a) The global (bulk) methylation level of samples. (b) Methylation levels of different methylation site types. (c) Trends in methylation levels of gene-bodies and flank regions.

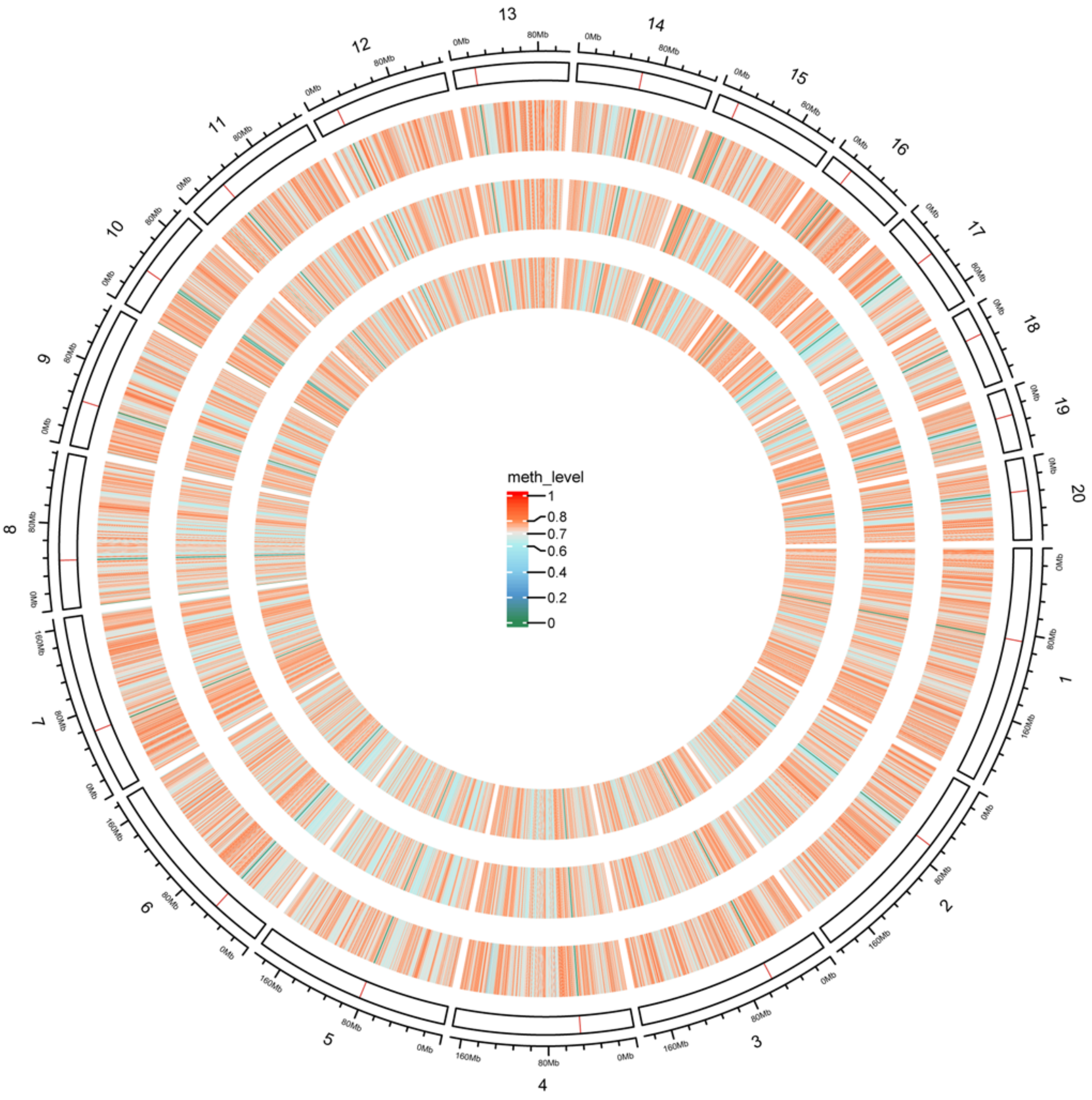

Supplementary Fig. S2 Mean methylation levels of three macaque species. The species represented from the outer circle to the inner circle are CR, TM and CE.The red line in the outermost box epresents the centromere.

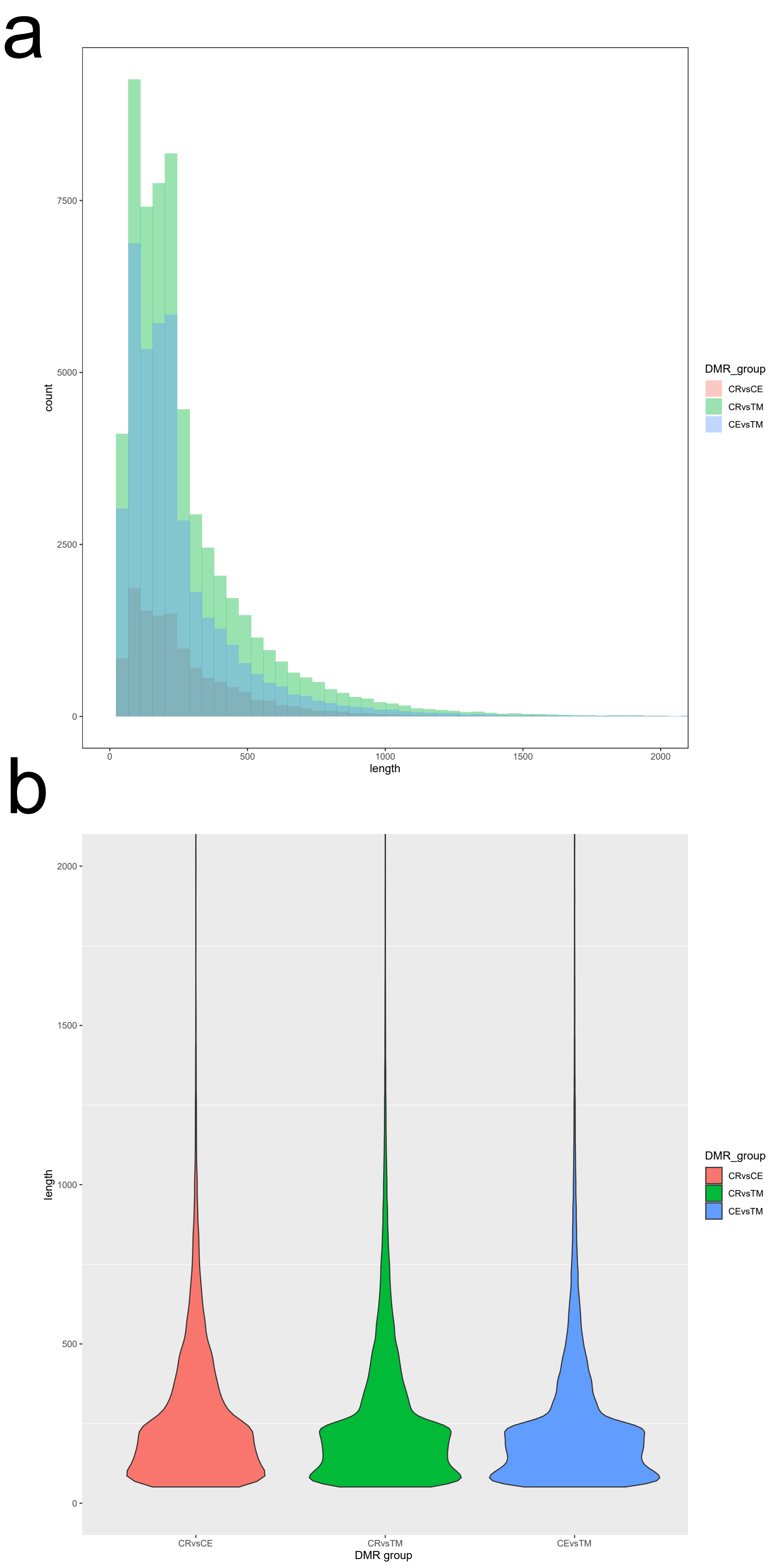

Supplementary Fig. S3 Statistical analysis of DMRs length. (a) Histogram of DMR length statistics, and length over 2 000 is omitted. (b) The violin figure of DMR length statistics.

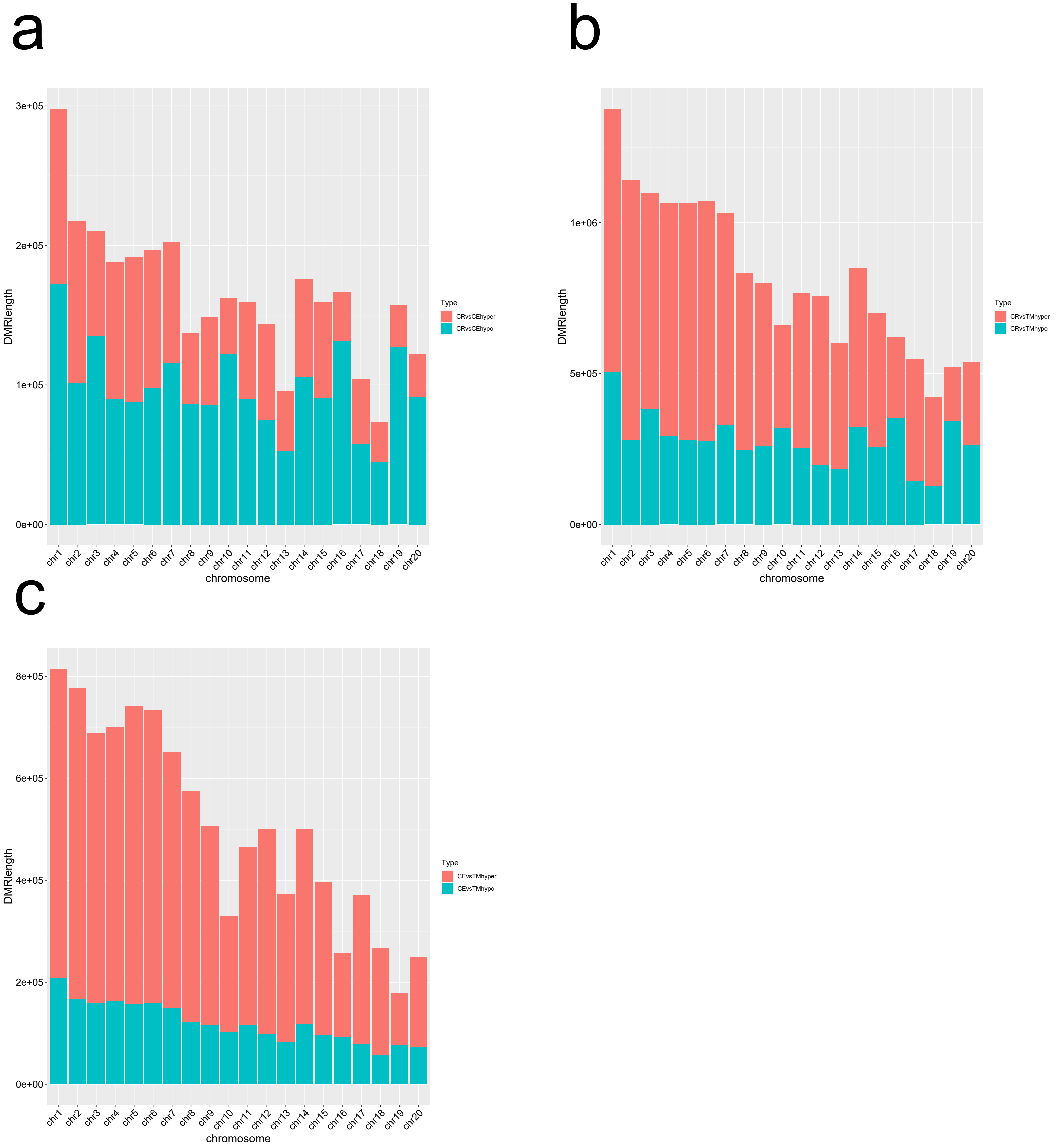

Supplementary Fig. S4 Distribution of DMR length on chromosomes. Different bar diagrams from (a) to (c) are CRvsCE, CRvsTM, and CEvsTM.

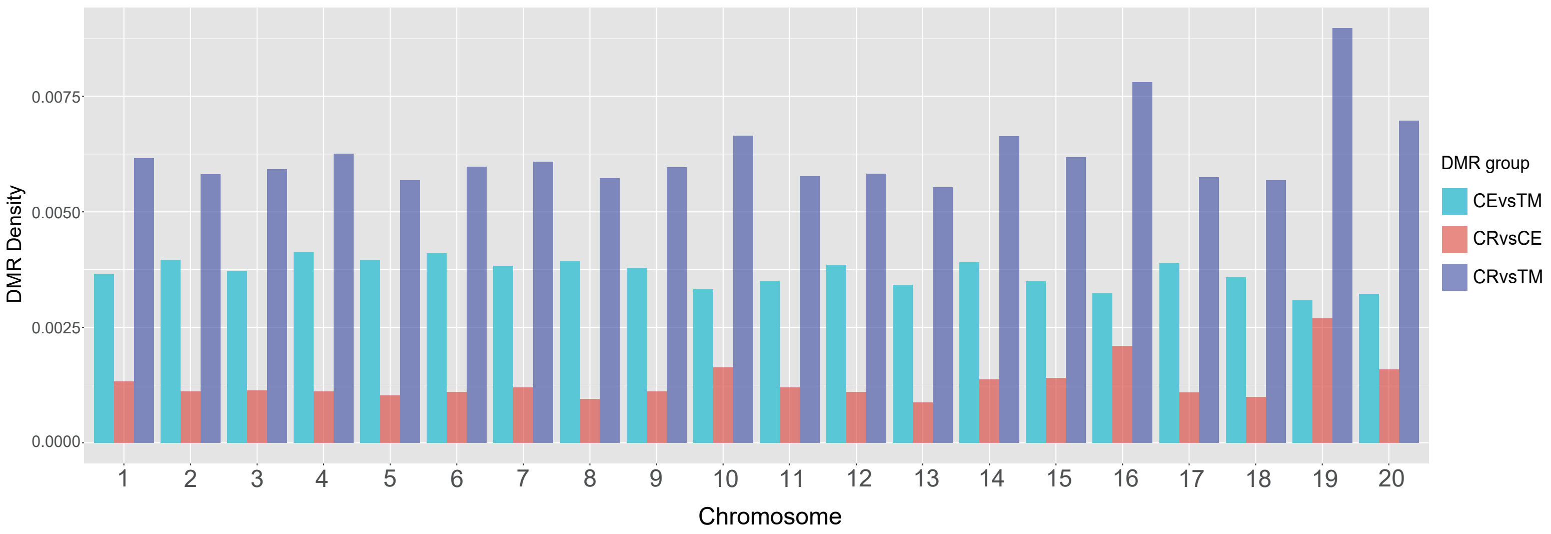

Supplementary Fig. S5 The density of DMRs at the chromosomal level.

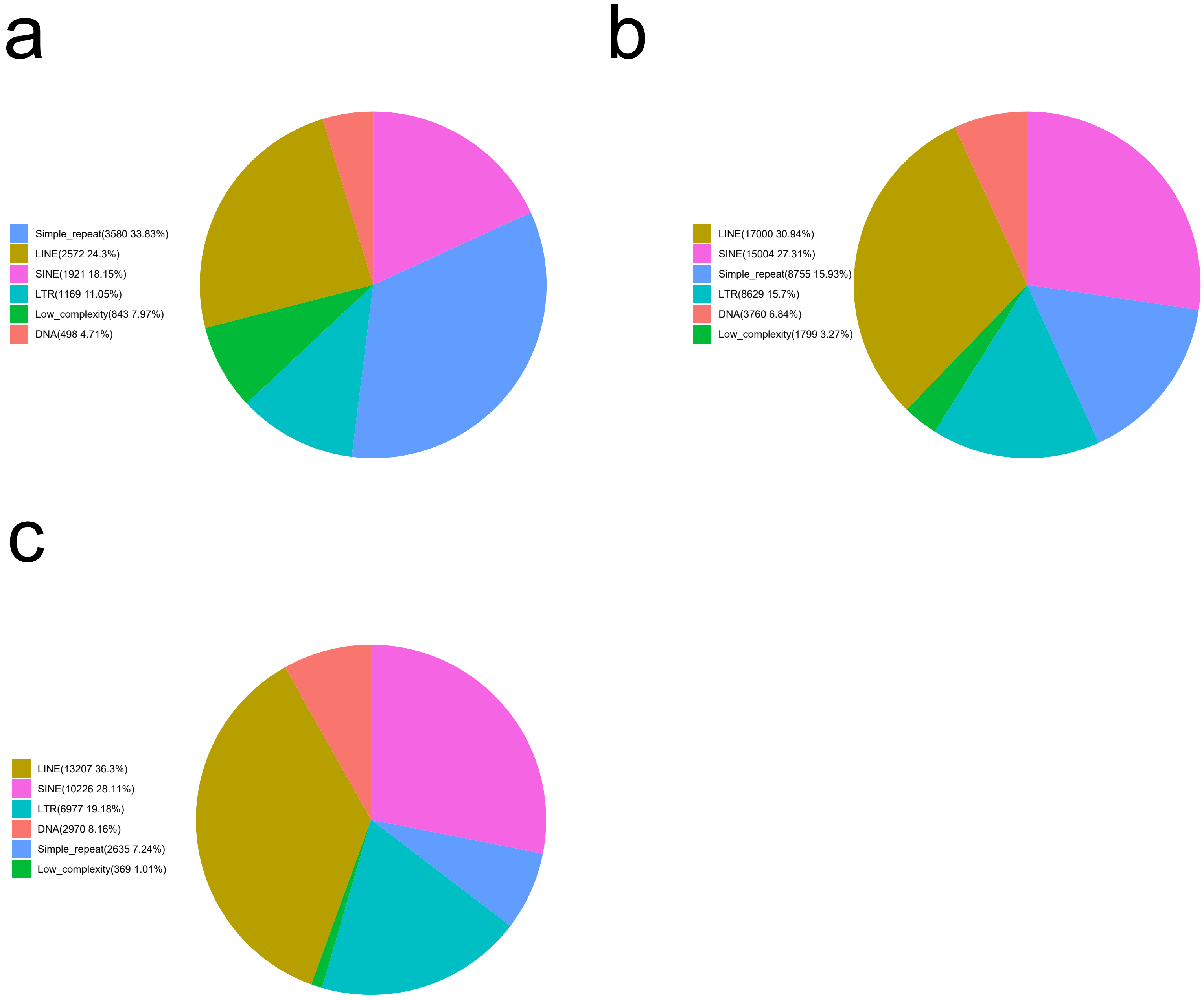

Supplementary Fig. S6 The percentage of genomic repeat elements overlapped with DMR. The different DMR groups from (a) to (c) are CRvsCE, CRvsTM, and CEvsTM.

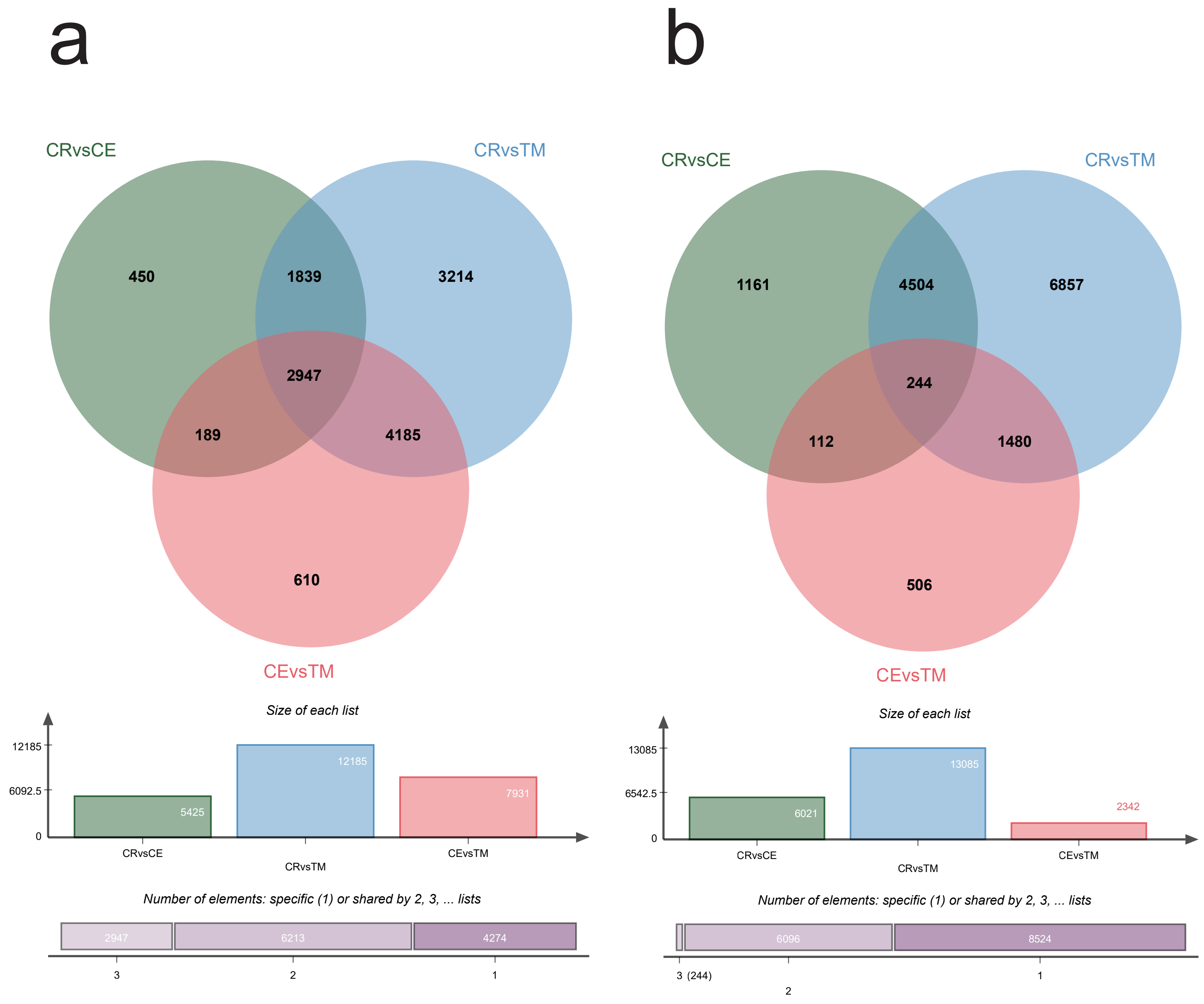

Supplementary Fig. S7 The overlap of (a) DMG and (b) DMP among three groups.

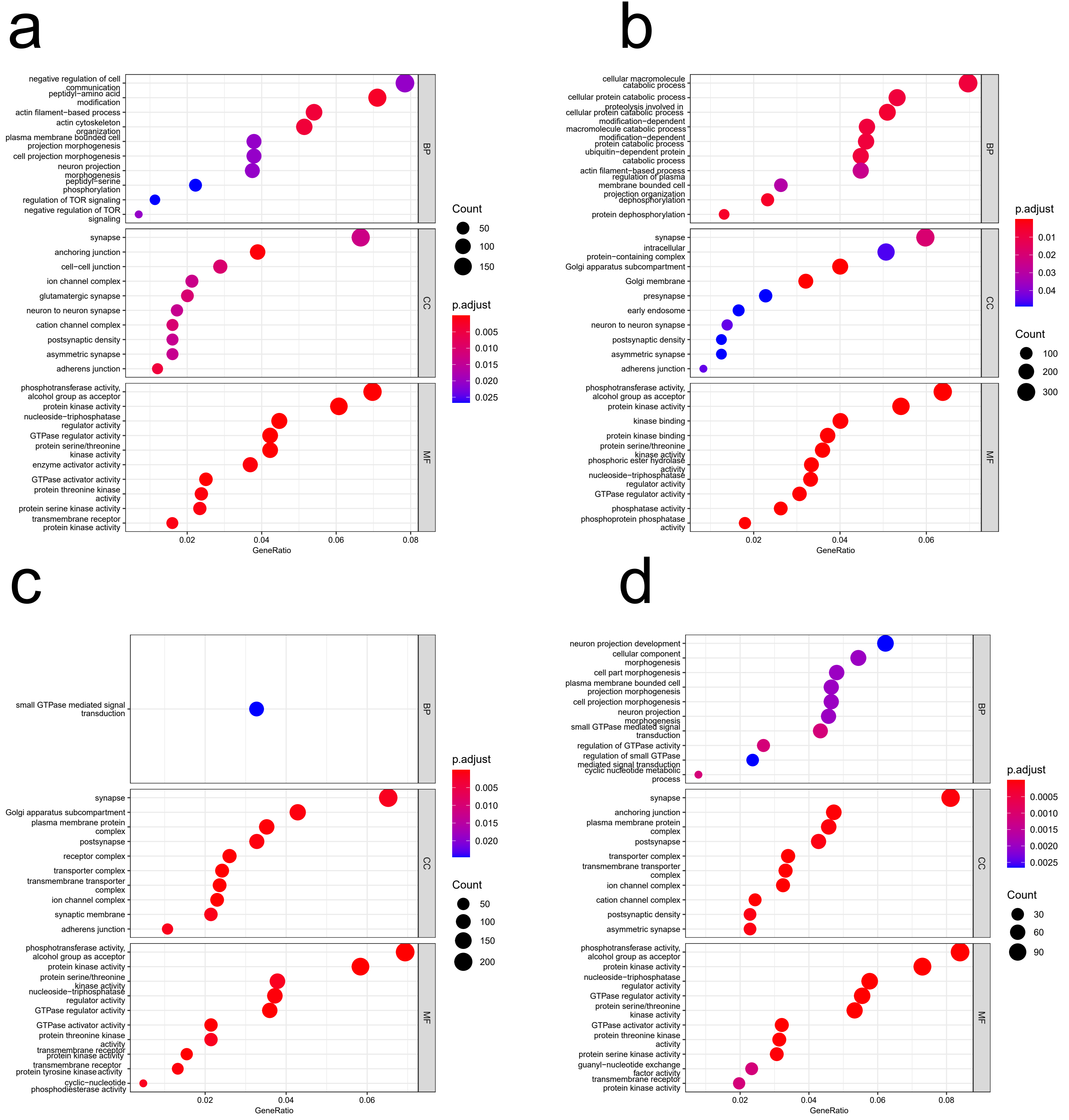

Supplementary Fig. S8 The GO enrichment results of differential methylation genes (DMGs). From (a) to (d) are the GO enrichment results of CRvsCE, CRvsTM, CEvsTM and intersection of DMG groups.

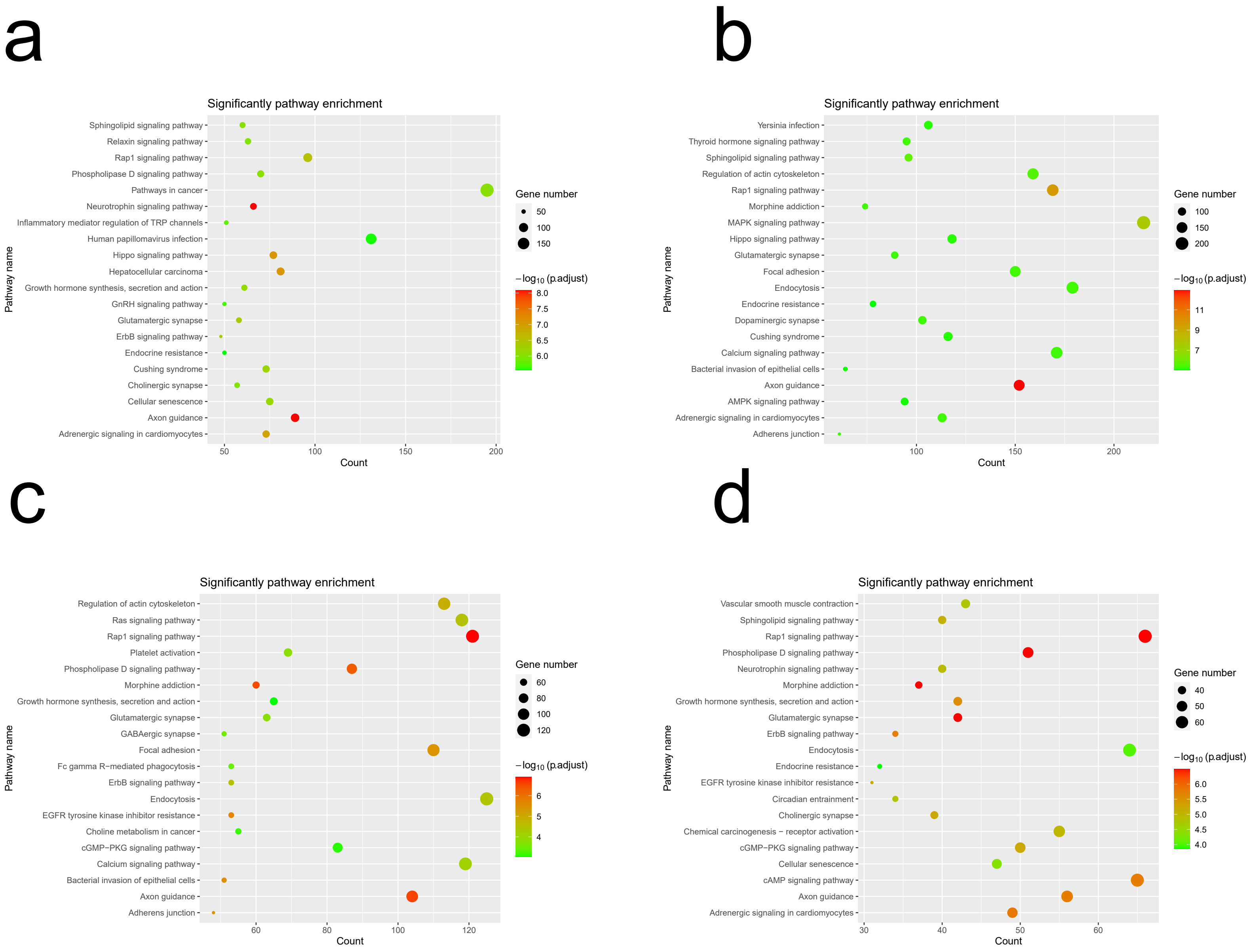

Supplementary Fig. S9 The KEGG pathways that differential methylation genes (DMGs) enriched. From (a) to (d) are the KEGG enrichment results of CRvsCE, CRvsTM, CEvsTM and intersection of DMG groups.

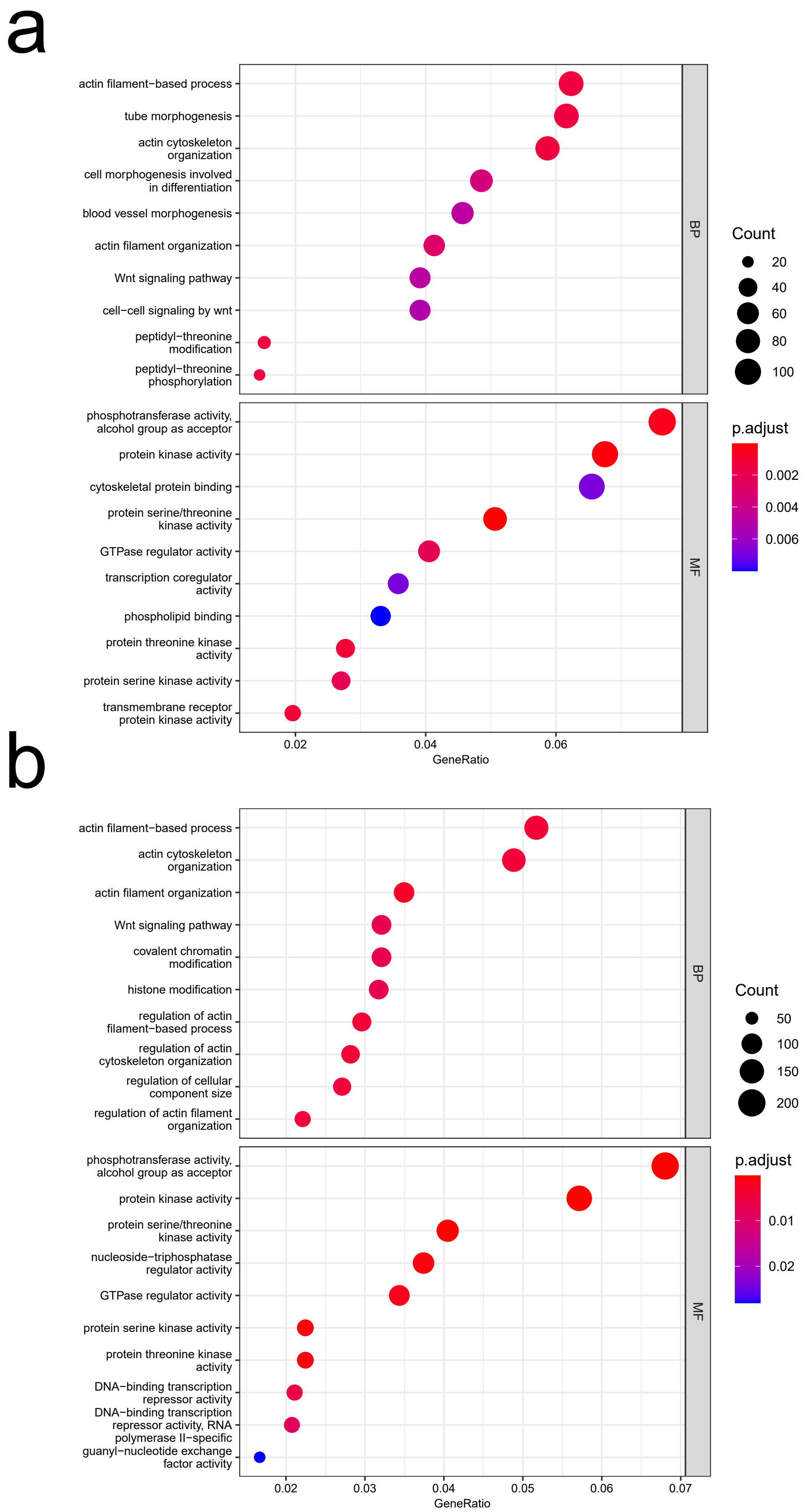

Supplementary Fig. S10 The GO terms that differential methylation promoters (DMPs) enriched. From (a) to (b) are the GO enrichment results of CRvsCE, CRvsTM.

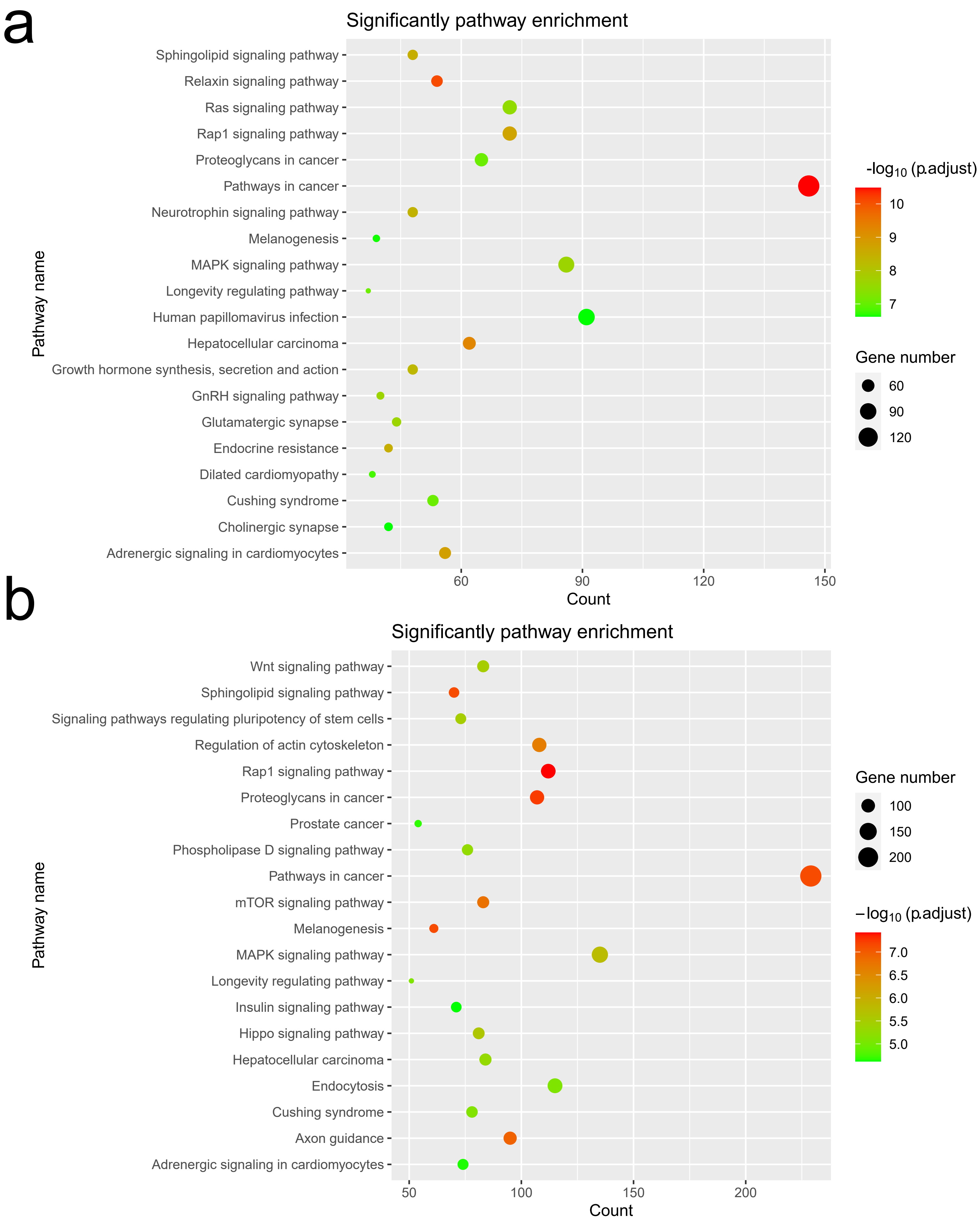

Supplementary Fig. S11 The KEGG pathways that differential methylation promoters (DMPs) enriched. From (a) to (b) are the KEGG enrichment results of CRvsCE, CRvsTM.

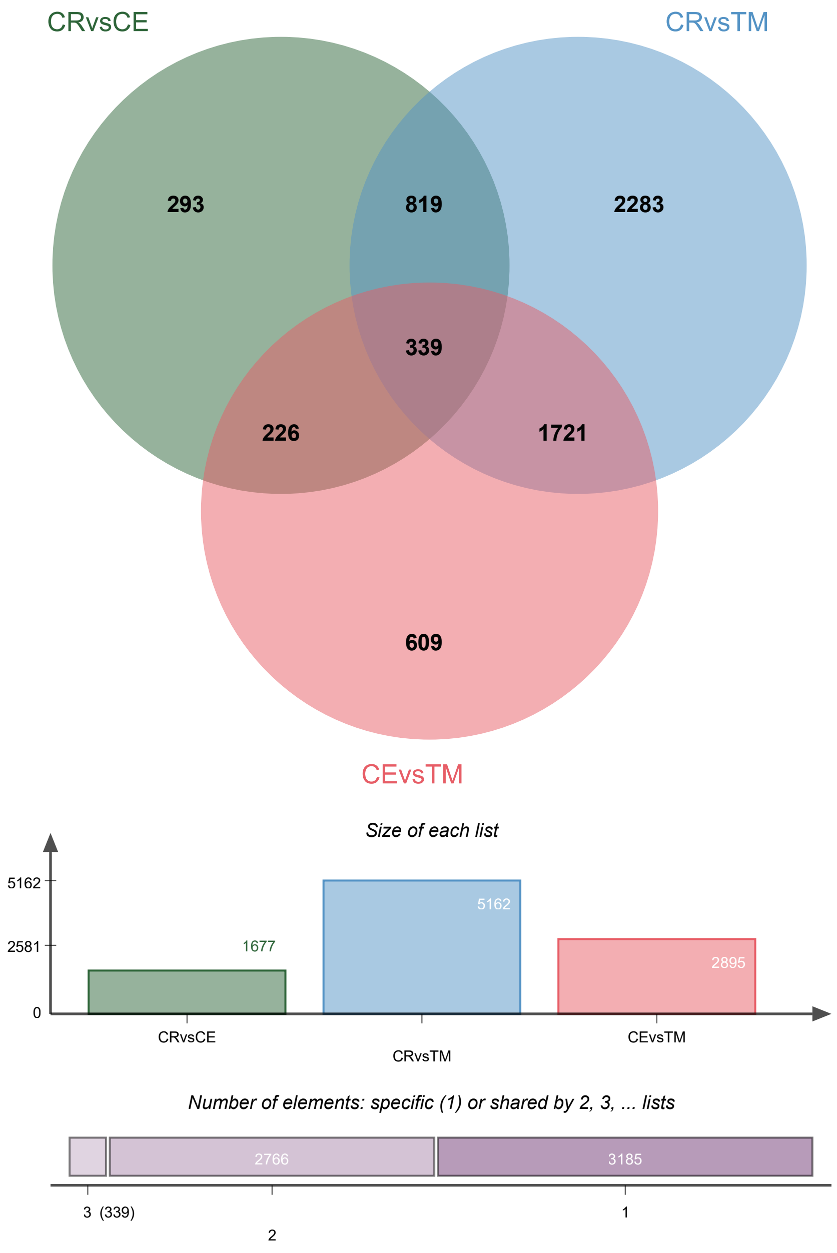

Supplementary Fig. S12 The overlop DEG of three groups.

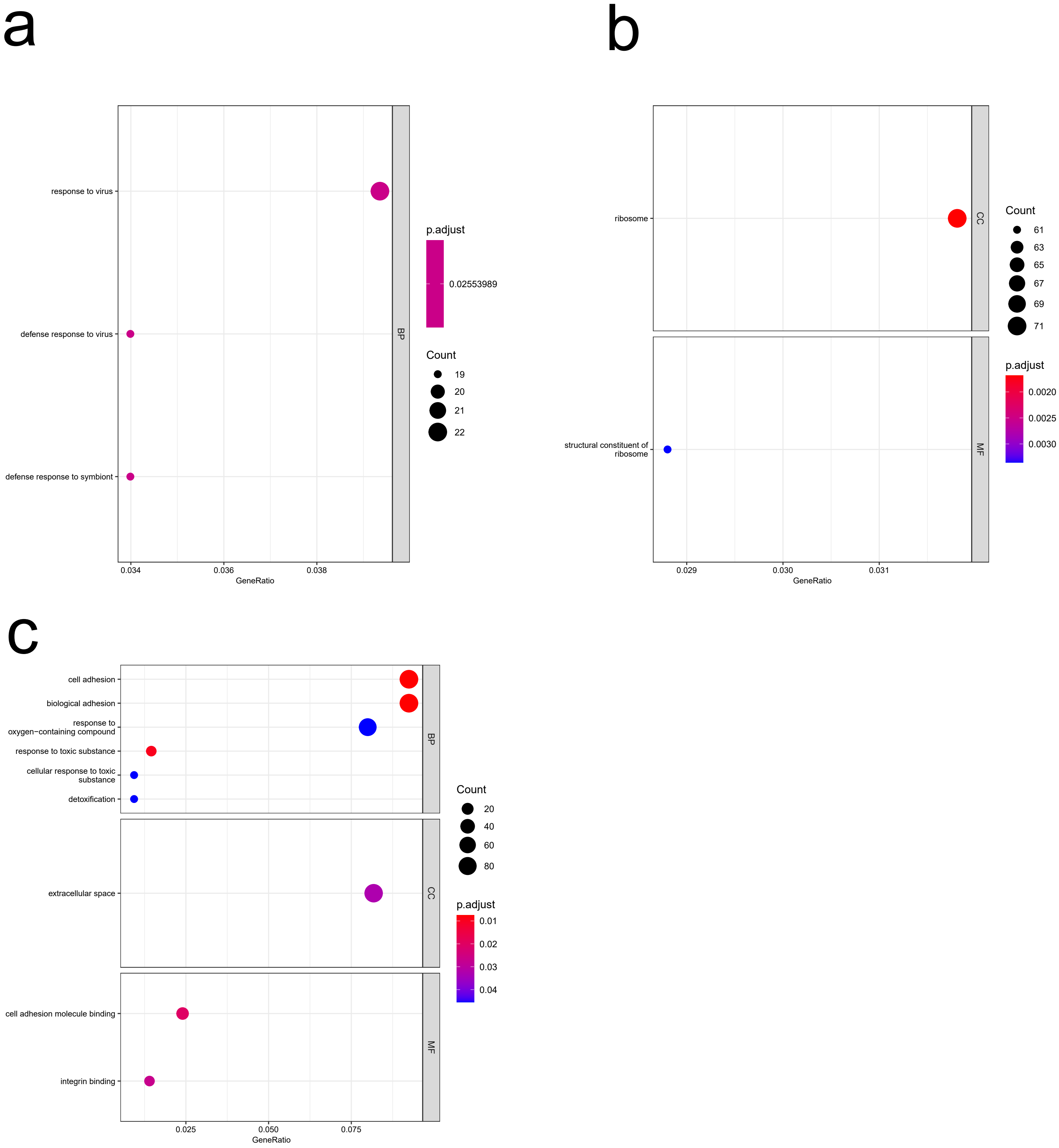

Supplementary Fig. S13 The GO terms that differentially expressed genes (DEGs) enriched. From (a) to (c) are the GO enrichment results of CRvsCE, CRvsTM and CEvsTM.

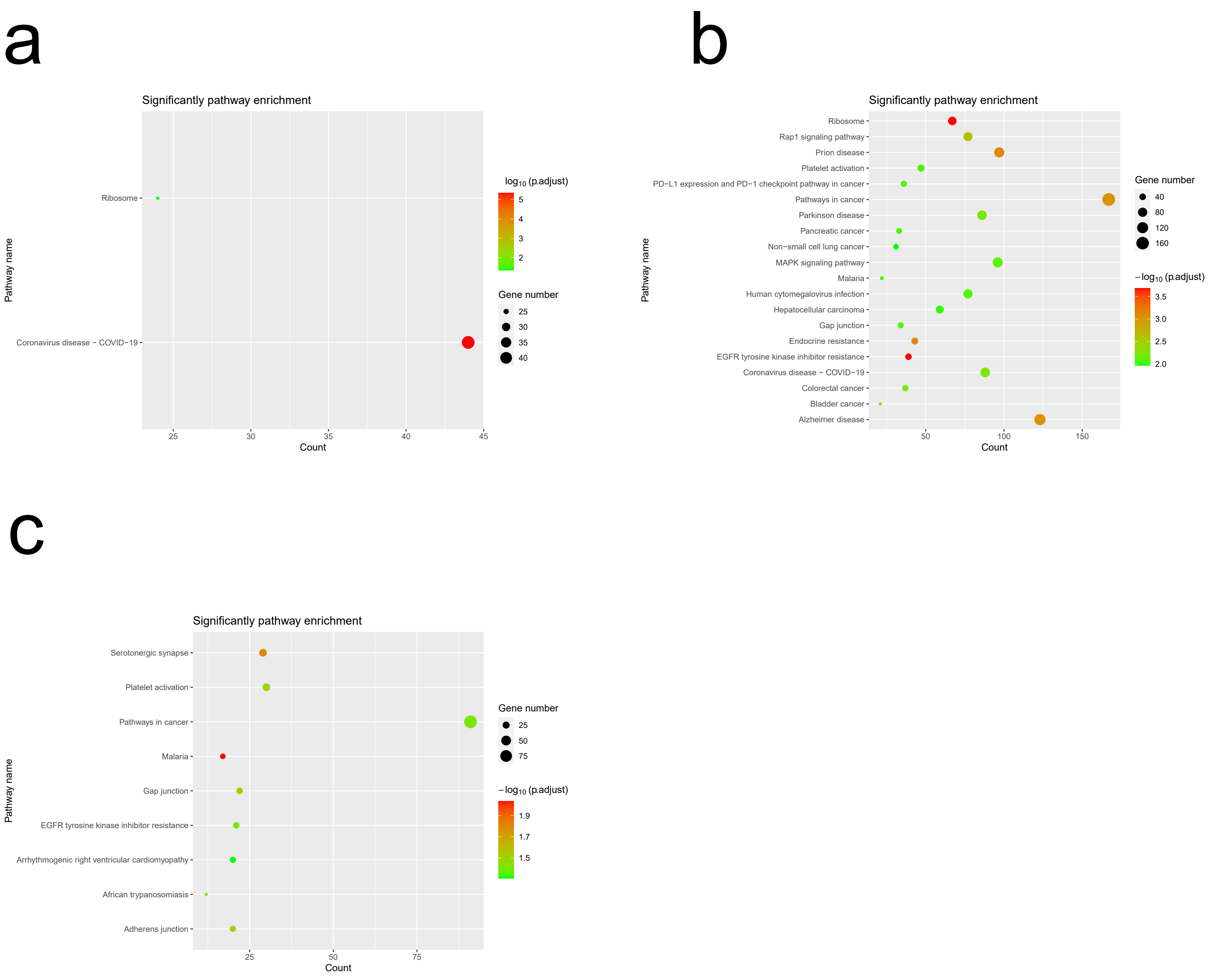

Supplementary Fig. S14 The KEGG pathways that differentially expressed genes (DEGs) enriched. From (a) to (c) are the KEGG enrichment results of CRvsCE, CRvsTM and CEvsTM.

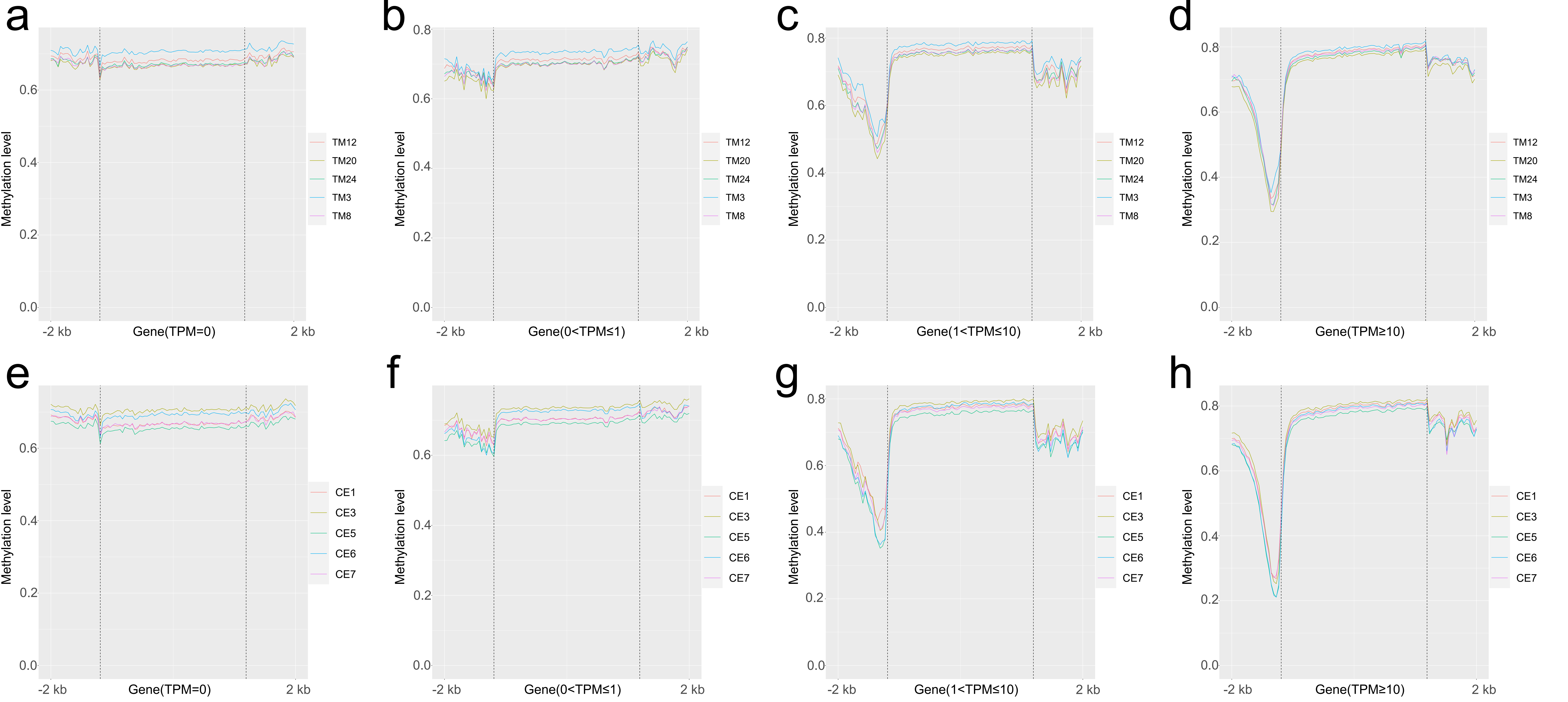

Supplementary Fig. S15 (a-h) Methylation trends of gene groups with different expression intensities in gene-body and flanking regions. In the figure, the corresponding data source from (a) to (d) is TM, and the corresponding data source from (e) to (h) is CE. The corresponding expression intensity groups were TPM = 0, 0 < TPM ≤ 1, 1< TPM ≤ 10 and TPM ≥ 10, respectively.

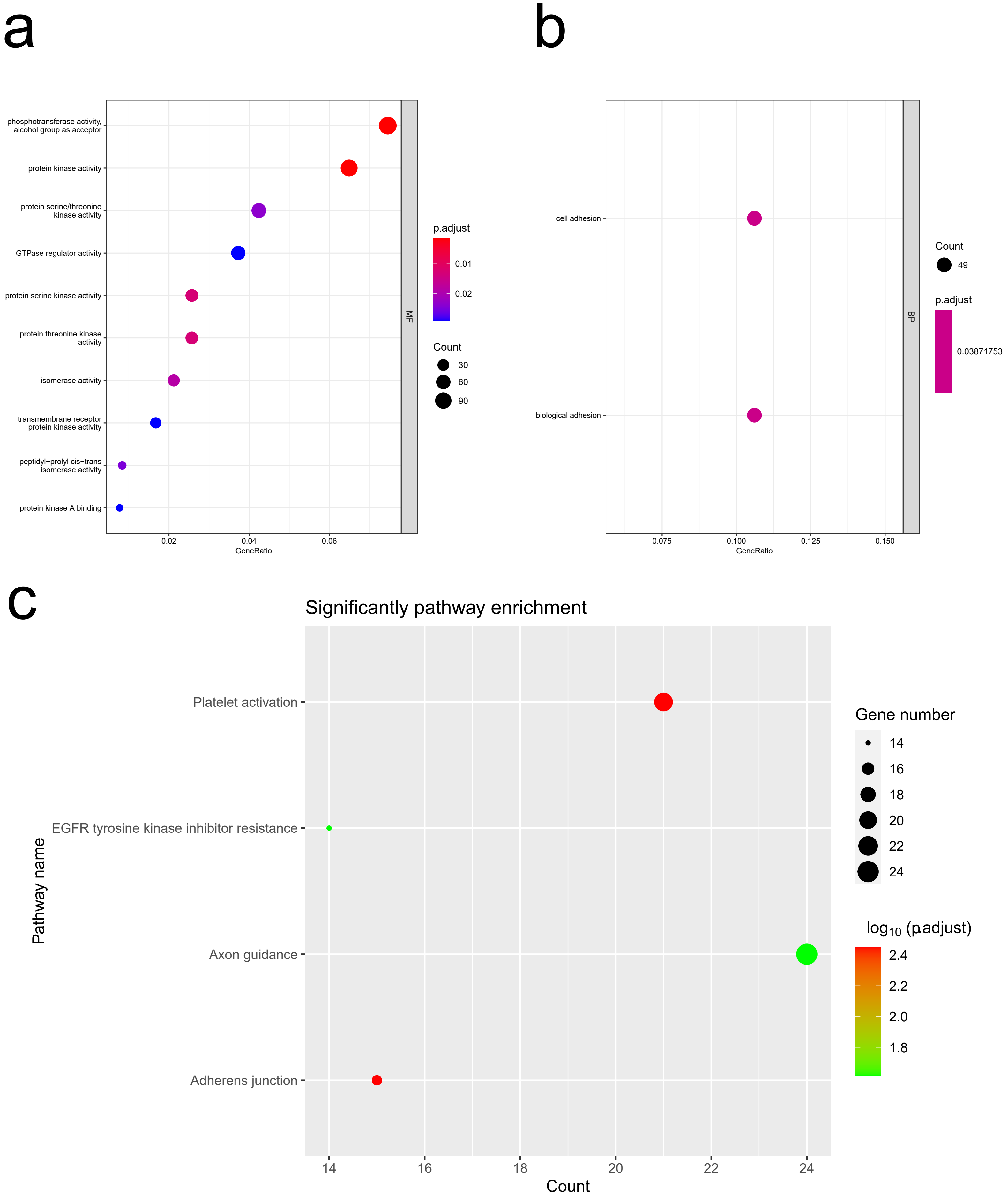

Supplementary Fig. S16 The GO terms and KEGG pathways that DMR-related DEGs enriched. From (a) to (b) are the GO enrichment results of CRvsTM and CEvsTM. (c) are the KEGG enrichment results of CEvsTM.

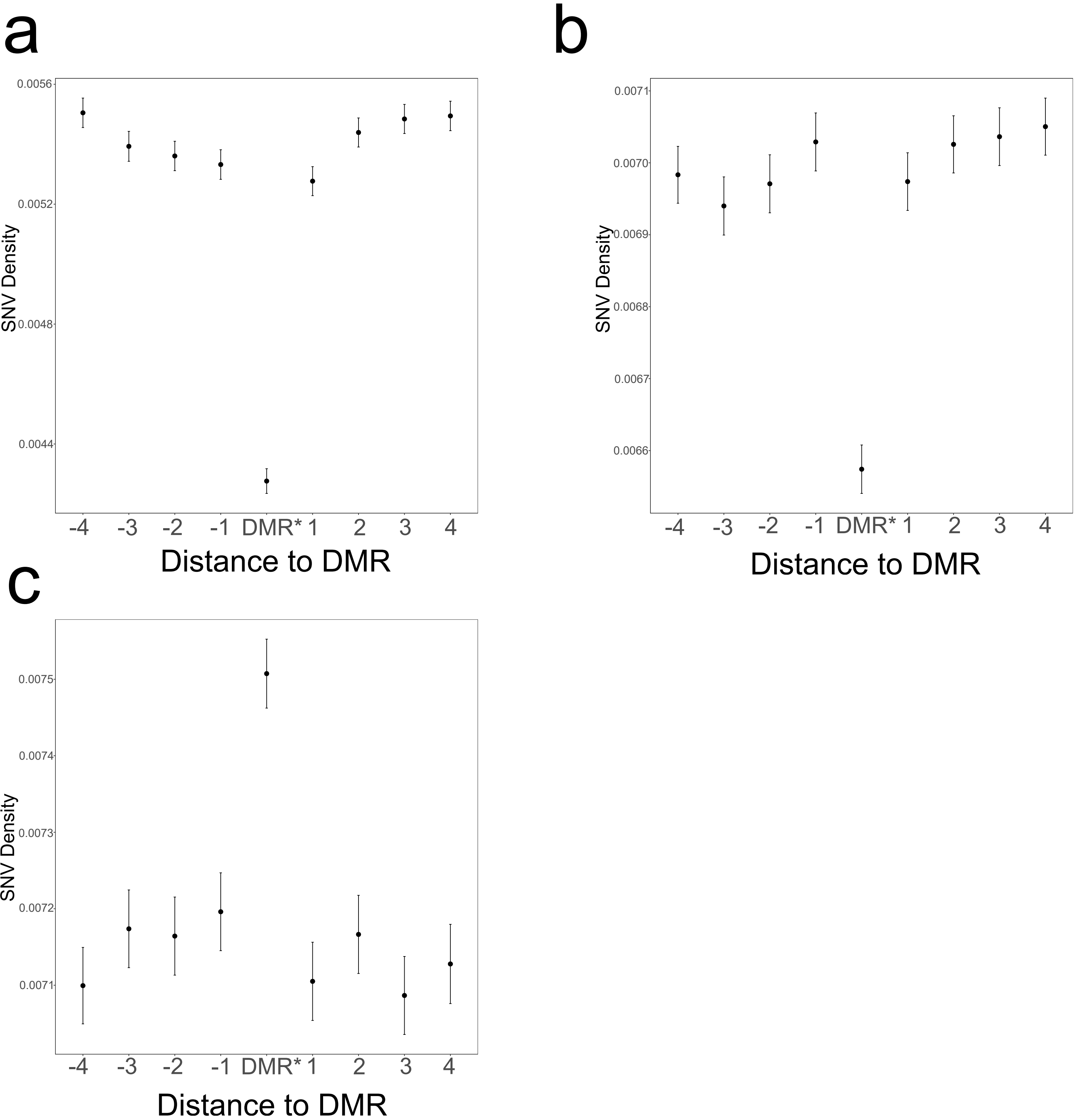

Supplementary Fig. S17 SNV density of DMR and its flanking regions. In the figure, the abscissa origin DMR* represents DMR and ± 1kb flanking sequence. ±1~±4 on the abscissa represents the 1kb region from DMR±1~± 4kb. The different SNV density groups from (a) to (c) are the hypomethylated DMRs of CRvsCE, CRvsTM, and CEvsTM.
