# Supplementary material for: DNA methylation and transcriptome analysis reveal epigenomic differences among three macaque species
Source: Evol Appl. 2023 Oct 12;17(2):e13604. doi: 10.1111/eva.13604 (PMC10853583; doi:10.1111/eva.13604)
Supplement: Supplementary file 3 — Appendix S3. [file EVA-17-e13604-s001.pdf]

Supplementary Materials and methods

1. Samples of BS-seq

Whole peripheral blood samples were collected by a professional expert and were approved by the Ethics Committee of Sichuan University and Sichuan provincial people’s hospital. Genomic DNA from macaque blood tissue was extracted following the Qiagen DNeasy Blood & Tissue Kit. DNA purity was checked using the NanoPhotometer spectrophotometer (IMPLEN, CA, USA). DNA concentration was measured using Qubit DNA Assay Kit in Qubit 2.0 Fluorometer (Life Technologies, CA, USA). A total of 5.2 micrograms genomic DNA spiked with 26ng lambda DNA were fragmented by sonication to 200-300bp with Covaris S220, which was added as a negative marker, followed by end repair and adenylation. Cytosine-methylated barcodes were ligated to sonicated DNA as manufacturer’s instructions. Then these DNA fragments were treated twice with bisulfite using EZ DNA Methylation-Gold TM Kit (Zymo Research).

Moreover, the resulting single-strand DNA fragments were PCR amplificated using KAPA HiFi HotStart Uracil + ReadyMix (2X). Library concentration was quantified by Qubit 2.0 Fluorometer (Life Technologies, CA, USA) and quantitative PCR and the insert size was checked on Agilent Bioanalyzer 2100 system. According to the manufacturer's instructions, the clustering of the index-coded samples was performed on a cBot Cluster Generation System using TruSeq PE Cluster Kit v3-cBot-HS (Illumia). After cluster generation, the library preparations were sequenced on an Illumina Hiseq 2000/2500 platform in Novogene (Beijing, China), and pair-end reads were generated. Image analysis and base calling were performed with the standard Illumina pipeline, and finally, paired-end reads were generated.

2. Samples of RNA-seq

We collected all peripheral blood samples during routine examination of healthy captive *M. fascicularis* in Sichuan Green-house Biotech Co., Ltd, Meishan, Sichuan. All *M. fascicularis* are kept in the same captivity. The Ethics Committee approved this study of the College of Life Sciences, Sichuan University (No. 20200529001); sample collection and use procedures are carried out according to the guidelines of the

management committee of experimental animals Sichuan Province, China (SYXK-Sichuan, 2019). Fresh blood samples were stored at room temperature (about 18-20 °C) for 4 h in a particular blood collection tube (PAX gene blood RNA tube), then transferred to-20 °C for 24 h, and then preserved at-80 °C until RNA extraction.

Sequencing of *M. fascicularis* samples involved using 3 μg RNA per sample as input material for the RNA sample preparations. The total RNA was extracted according to the manufacturer's manual of the PAX gene blood RNA kit, and the quality of RNA was evaluated by Agilent 2100 biological analyzer (Agilent Technologies, Santa Clara, CA). The total RNA was treated with GlobinZero kit (Epicentre, Illumina, Madison, WI) and purified by modified Qiagen RNeasy MinElute (Qiagen Inc., Valencia, CA) purification procedure or ethanol precipitation. Samples with RIN (RNA integrity number) greater than 7.5 are used for library construction, sequencing, and real-time quantitative PCR (RT-qPCR). Library preparation and all sequencing operations are carried out following the manufacturer's instructions. We used a strand-specific kit from Epicentre (ScriptSeq v2 Library Prep kit, Illumina, Madison, WI) with ScriptSeq Index PCR primers (Epicentre, Illumina, Madison, WI) to convert single-strand RNA into cDNA by reverse transcription PCR. In short, each cDNA sample is ultrasonically processed into 300-500bp fragments for double-terminal library generation. We used the Illumina NovaSeq 6000 to sequence all the libraries with a paired-end sequencing length of 150 bp (PE150) at Novogene (Beijing, China).
